# *LactococcusCeduovirus* Phages Isolated from Industrial Dairy Plants—From Physiological to Genomic Analyses

**DOI:** 10.3390/v12030280

**Published:** 2020-03-03

**Authors:** Magdalena Chmielewska-Jeznach, Jacek K. Bardowski, Agnieszka K. Szczepankowska

**Affiliations:** Institute of Biochemistry and Biophysics, Polish Academy of Sciences, Pawinskiego 5a, 02-106 Warsaw, Poland; mchjeznach@ibb.waw.pl (M.C.-J.); jacek@ibb.waw.pl (J.K.B.)

**Keywords:** bacteriophage, *Lactococcus*, *Ceduovirus*, dairy, host range, comparative genome analysis, origin of replication

## Abstract

*LactococcusCeduovirus* (formerly c2*virus*) bacteriophages are among the three most prevalent phage types reported in dairy environments. Phages from this group conduct a strictly lytic lifestyle and cause substantial losses during milk fermentation processes, by infecting lactococcal host starter strains. Despite their deleterious activity, there are limited research data concerning *Ceduovirus* phages. To advance our knowledge on this specific phage group, we sequenced and performed a comparative analysis of 10 new *Lactococcus* *lactis* *Ceduovirus* phages isolated from distinct dairy environments. Host range studies allowed us to distinguish the differential patterns of infection of *L. lactis* cells for each phage, and revealed a broad host spectrum for most of them. We showed that 40% of the studied *Ceduovirus* phages can infect both *cremoris* and *lactis* strains. A preference to lyse strains with the C-type cell wall polysaccharide genotype was observed. Phage whole-genome sequencing revealed an average nucleotide identity above 80%, with distinct regions of divergence mapped to several locations. The comparative approach for analyzing genomic data and the phage lytic spectrum suggested that the amino acid sequence of the *orf8*-encoded putative tape measure protein correlates with host range. Phylogenetic studies revealed separation of the sequenced phages into two subgroups. Finally, we identified three types of phage origin of replication regions, and showed they are able to support plasmid replication without additional phage proteins.

## 1. Introduction

*Ceduovirus* phages infecting *Lactococcus lactis* starter strains are one of the three most prominent phage species in the dairy environment worldwide [1,2]. Phages classified to this type belong to the *Siphoviridae* family of the *Caudovirales* order, and are characterized by prolate heads and long non-contractile tails [3]. Members of the *Ceduovirus* (or prolate) group have a double-stranded (ds) DNA genome and conduct an obligatory lytic life cycle. Despite the acknowledged ubiquity of *Ceduovirus* phages in milk plants, there is a significant deficiency in the amount of experimental data concerning this group of phages. This stands in strong contrast to the well-characterized and abundantly sequenced *Lactococcus Skunavirus* (formerly 936-type or sk1*virus*) type [4,5,6]. Until now, less than 40 complete genome sequences of *Ceduovirus* phages were available in databases [7,8,9,10,11,12,13]. Based on previous studies, involving genome sequencing, *Ceduoviruses* were determined to represent a genetically similar phage group. Their genomes are organized into two divergently transcribed blocks comprising early and late genes, respectively, separated by a non-coding region containing the origin of phage replication [7,8,9,14]. In addition to the overall high genome conservation among prolate phages, certain regions of divergence can be observed, including (i) the early transcribed region (due to presence/lack of individual open reading frames—*orfs*), (ii) in the late region, particularly within structural genes implicated in host range determination, and (iii) in the non-coding intergenic region containing the origin of phage replication [8,9,14]. Determining the correlations between genomic data and host range patterns of *Lactococcus* phages has been the main objective of numerous studies, and is an important aspect in understanding the mechanisms of phage prevalence in the dairy environment [9,15,16,17]. Studies conducted on c2 and bIL67, the two model *Ceduovirus* phages, have appointed the respective gene regions *l14-l15-l16* (locus_tag: c2p36-c2p37-c2p38) and *orf34-orf35-orf36* (locus_tag: gp04-gp03-gp02) as host range determinants [9,15]. These genes are suggested to encode structural proteins, possibly tail fibers, but so far this has not been experimentally confirmed. Analysis of ORF*ll5*_*c*2_/ORF35_bIL67_ and ORF*l16*_*c*2_/ORF36_bIL67_ using the HHPRED tool predicted structural similarity to proteins with carbohydrate-binding domains (5E7T_B for ORF*ll5*_*c*2_/ORF35_bIL67_ and 3P6B_B for ORF36_bIL67_ and 1GU3_A for ORF*l16*_*c*2_), which assumes their engagement in adhesion to host cells. In turn, both, phage bIL67 *orf34* and phage c2 *l14* are annotated as encoding hypothetical proteins. By analogy, genes of other *Ceduovirus* phages localized in the same genetic context as *l14-l15-l16* and *orf34-orf35-orf36* are also expected to be crucial in defining their lytic spectrum. Additionally, genes *l10* (locus_tag: c2p32) of phage c2 and *orf31* (locus_tag: bIL67_gp07) of phage bIL67 have been reported to be implicated in host cell infection [8,15,18].

Efficient infection of *L. lactis* strains by *Ceduovirus* phages also relies on bacterial factors. Initiation of *Ceduovirus* phage infection is described as a two-step process relying on (i) reversible adsorption to the bacterial cell wall polysaccharide (CWPS) followed by (ii) irreversible binding to a proteinaceous receptor [19,20,21]. A study by Oliveira and co-workers [10] on 21 *Ceduovirus* phages determined their strong preference for bacterial hosts with the type C CWPS genotype. This suggests that although the interaction with the host CWPS is reversible, it may be crucial in the initial step of infection, ensuring a high rate of phage adsorption and effective propagation. Among the features differentiating the otherwise conserved *Ceduovirus* phage group is the ori region, which is localized between the early and late transcribed gene clusters [22]. Multiple sequence alignments of *Ceduovirus* phage genomes have allowed identification of three distinct ori groups: bIL67-type, c2-type, and 923-type [14]. A typical feature of all ori types is a non-coding sequence flanked by P_E1_ and P_E2_—the first two promoters from the early transcribed region. The nucleotide sequence between these two promoters is the major varying feature of *Ceduovirus* phage ori types. Based on studies of phage c2, the P_E1_ promoter was found to be involved in production of a non-coding transcript (P_E1_-T) which may have a crucial role in initiating phage replication in a transcription-mediated fashion [23,24]. Both the length and the sequence and/or structure of P_E1_-T were suggested to be crucial factors of each ori type—chimeric origins were non-functional [25].

We sequenced and characterized 10 *Lactococcus Ceduovirus* phages isolated from distinct dairy whey samples derived from disturbed production processes in Poland. By analyzing the phage infection patterns, a correlation with the subspecies of the *L. lactis* hosts and their cell wall polysaccharide (CWPS) profiles was observed. Comparative analysis of the sequenced phage genomes allowed us to establish the relatedness and determine several distinguishing features with respect to other *Lactococcus Ceduovirus* phages. Phylogeny studies revealed clustering of the analyzed phages with other *Ceduovirus* phages into two major subgroups, irrelevantly of their geographical settings. Moreover, by using comparative analysis we identified the putative origins of replication of the studied phages and classified them into one of the previously identified *Ceduovirus* phage ori groups (c2-, bIL67-, or 923-like). Functional studies confirm the ability of the phage oris to support plasmid replication in *L. lactis* strains.

## 2. Materials and Methods

### 2.1. Bacterial Strains, Bacteriophages, and Culture Conditions

Bacterial strains and bacteriophages used in study are listed in Table 1. *Lactococcus lactis* strains were grown at 30 °C on M17 broth (Oxoid) supplemented with 0.5% glucose (GM17) or on GM17 solid-agar (1.5%). When needed, GM17 plates were supplemented with chloramphenicol (Cm) at 10 µg mL^−1^. GM17 medium for phage propagation was supplemented with 10 mM CaCl_2_ (GM17-CaCl_2_). *Escherichia coli* strain was grown at 37 °C in Luria-Bertani (LB) broth with shaking or on LB solid-agar (1.5%). When needed, LB plates were supplemented with tetracycline (Tet) at 8 µg mL^−1^ and ampicillin (Amp) at 100 µg mL^−1^. Bacteriophages derived from whey samples collected from dairy plants over a period of 4 years from eight locations across Poland.

### 2.2. Bacteriophage Propagation

*L. lactis* host strains were cultured in 200 mL of GM17-CaCl_2_ broth at 30 °C until OD_600_ 0.15–0.2. After reaching the desired OD_600_ the culture was infected by adding 0.2 mL of high titer phage lysate (≥10^9^ plaque forming units, PFU, per mL) and further incubated at 30 °C until complete lysis (up to 2 h). The lysate was filtered using 0.45 μm filters (Stericup Millipore) and kept at 4 °C until further use.

### 2.3. Electron Microscopy

Phage lysates were concentrated for electron microscopy by centrifugation (14,000 rpm, 4 °C for 4 h), the pellet was washed with 100 mM ammonium acetate, then centrifuged (14,000 rpm, 4 °C for 1 h) three times and stained using phosphoric potassium tungstate (2% *w*/*v*) on Formvar-coated copper grid. Micrographs were performed using the JEM 1400 (JEOL Co., Japan) electron microscope equipped with energy-dispersive full range X-ray microanalysis system (EDS INCA Energy TEM, Oxford Instruments, UK), tomographic holder and 11 Megapixel TEM Camera MORADA G2 (EMSIS GmbH, Germany) in the Laboratory of Electron Microscopy at the Nencki Institute of Experimental Biology (Warsaw, Poland).

### 2.4. Host Range Studies

Phage infection specificity was tested against a collection of 57 *L. lactis* strains, comprising industrial (50), environmental (4), and laboratory (3) isolates by double-layer plate method, as described elsewhere [30]. Phage titer (PFU per mL) was determined by mixing the diluted phage lysates (10^−2^–10^−8^; 100 µL) with overnight-grown bacteria (200 µL) and plating in GM17 soft-agar (0.75%) on GM17-CaCl_2_ plates. After overnight (o/n) incubation at 30 °C, the number of plaques for each dilution was counted and the phage titer calculated. The same approach was applied for host range studies. Phage-sensitive strains were detected after o/n incubation at 30 °C by screening plates for the presence of lytic zones.

### 2.5. One-Step Growth Assay

One-step growth studies were done according to a previously established protocol [31]. Phage titer (PFU per mL) was calculated by counting the number of visible lysis zones after o/n incubation at 30 °C. Burst size was determined by dividing the final average count of liberated phage particles (at plateau) by the initial average count of infected bacterial cells. Time prior to the release of infection particles was established as the latent period. Burst time was inferred directly from the one-step growth curve as the time needed for the phage to complete one cycle. The assay was repeated three-fold.

### 2.6. PCR-Based Typing

Primers used in this study are listed in Table 2. Phages were assigned to the *Ceduovirus* phages based on PCR reaction using *Ceduovirus*-specific primers (c-2for/c-2rev) and phage lysate (1 μL) as template. Phage c2 and phage sk1 lysates served in the assay as the positive and negative control, respectively. PCR products were run on a 0.8% (*w*/*v*) agarose gel in 1xTAE (Tris acetate-EDTA) buffer stained with ethidium bromide and analyzed under UV light using the G:BOX instrument (Syngene).

Cell wall polysaccharide typing was done as described elsewhere using primers specific for each CWPS (A, B, or C) (Table 2) and a single colony of each tested *L. lactis* strain as template [32]. A control reaction was run for each strain using the CTL_for/CTL_rev primers.

### 2.7. Phage Genome Sequencing

Phage genomic DNA was isolated from 200 mL of filtered phage lysates as described previously [33] and stored at 4 °C until further use. Sequencing of phage genomes was performed in the Laboratory of DNA Sequencing and Oligonucleotide Synthesis (IBB PAS) using Illumina Sequencing Technology. DNA was sheared mechanically using Covaris instrument (Covaris, Inc., Woburn, MA, USA) and TruSeq-like libraries were constructed using KAPA Library preparation kit (KAPA/Roche, Basel, Switzerland) according to the manufacturer’s instructions. Phage genomes were sequenced in paired-end mode (v3, 600 cycle chemistry kit) using the MiSeq instrument (Illumina, San Diego, CA, USA). Obtained sequence reads were filtered by quality using the fastp toolkit [34]. The Newbler version 3.0 software program (454 Life Sciences, Branford, CT, USA) was used for sequence assembly. Sequencing coverage depth was 100–120X for each assembled sample. Ambiguous sequence regions were amplified by PCR with specific primers and re-sequenced using the BigDye Terminator Mix v. 3.1 chemistry (Applied Biosystems, Foster City, CA, USA) and the ABI3730xl Genetic Analyzer (Life Technologies, Carlsbad, CA, USA). Terminal repeat regions were re-sequenced by Sanger sequencing with phage DNA as a template and primers complementary to the non-redundant parts at the end of the analyzed region. All sequence errors and mis-assemblies were corrected using the Seqman software (DNAStar, Madison, WI, USA). Assembled genomes were annotated automatically using the Rapid Annotation Subsystem Technology (RAST) server [35].

### 2.8. Bioinformatic Analyses

Further functional annotations of predicted amino acid sequences were performed manually by employing the BLASTp algorithm (cut-off E-value 0.0001) [36] and on-line programs accessible through the SIB ExPASy Bioinformatics Resource Portal (https://www.expasy.org) (UniProtKB, PROSITE, SwissModel) and MPI Bioinformatics Tool Kit (HHPRED with pdb, pfam, prk, tigr, and cogs databases) (https://toolkit.tuebingen.mpg.de/) [37,38]. Structural homologs were additionally searched using Phyre2 [39]. The ClustalOmega tool at default settings implemented by the EMBL-EBI website was used for comparative alignment of amino acid sequences predicted for each locus [40]. All-against-all comparison of phage nucleotide sequences was done by multiple pairwise alignment using BLAST (bl2seq) [36]. Schematic representation of the genomes was obtained using the Easyfig 2.1 software [41]. Multiple sequence alignments for proteins annotated as putative DNApol (with RecA-like NTPase superfamily domain cl28885), tail fiber (GP2 and GP4), major capsid (GP14), holin, lysin, and ERF-like (essential recombination function) recombinase were calculated with Mafft [42] and trimmed with trimAl [43] to remove poorly aligned regions. For each protein, a maximum likelihood tree was computed using PhyML [44], based on the model of evolution estimated individually for each multiple sequence alignment with ProtTest [45]. All trees were further used for building the supertree with URec [46]. Phylogenetic trees were visualized with iTol [47].

### 2.9. Construction of Phage Ori+ Plasmids

Phage-derived DNA fragments containing putative ori sequences representing c2-, bIL67-, and 923-like ori groups were amplified by PCR technique using suitable primers (Table 2) and respective phage (94p4, 27, p6/4 and c2) lysates as templates. The 5′ extremities of the primers were modified by adding sequences recognized by either *Eco*RI or *Eco*RV (forward) and *Hind*III (reverse) restriction enzymes. Amplified DNA fragments were cut and ligated with the pUN121 vector digested by the same respective enzymes and introduced into *E. coli* 2566 cells by means of electroporation. Transformants were selected on LB plates containing Tet (8 µg mL^−1^) and Amp (100 µg mL^−1^). The resultant recombinant plasmids (pUN121:ori27, pUN121:ori94p4, pUN121:orip6/4, pUN121:oric2) were confirmed by colony PCR using pUN121_F/ pUN121_R primers. The vector carries a tetracycline (Tet) and ampicillin (Amp) resistance markers and is able to replicate in *E. coli* cells but lacks a functional ori and an appropriate selection marker for *L. lactis* cells. To select for the ori-carrying plasmids in *L. lactis* cells, a chloramphenicol resistance marker (catR) was introduced. For this, the *catR* gene functional in *L. lactis* cells was amplified using the pG+host3 vector as a template and suitable primers carrying SalI and BamHI sites at the 5′ extremities (Table 2). The SalI/BamHI-digested *catR* product was ligated with the pUN121:ori27, pUN121:ori94p4, pUN121:orip6/4 and pUN121:oric2 plasmids cut with the same enzymes and introduced by electroporation into *E. coli* 2566 cells. Transformants were analyzed by colony PCR using primers pUN121_catR_F/pUN121_catR_R and the cloned fragments were verified by DNA sequencing.

### 2.10. Functional Analysis of Putative Phage Ori Regions

To confirm the ability of the cloned putative oris to carry out plasmid replication, *L. lactis* IL1403 and MG1363 cells were electroporated with 1000 ng of pUN121:catR:ori27, pUN121:catR:ori94p4, and pUN121:catR:orip6/4 plasmid DNA. Transformants were selected on GM17 plates supplemented with Cm at 10 µg mL^−1^. Relative transformation efficiency was calculated in respect to transformants carrying the pG+host3 plasmid which was used as a control for competence check. The pUN121:catR:oric2 vector carrying the previously identified phage c2 ori served for the method control.

## 3. Results

### 3.1. Phage Classification and Morphological Analysis

**Figure 1:**
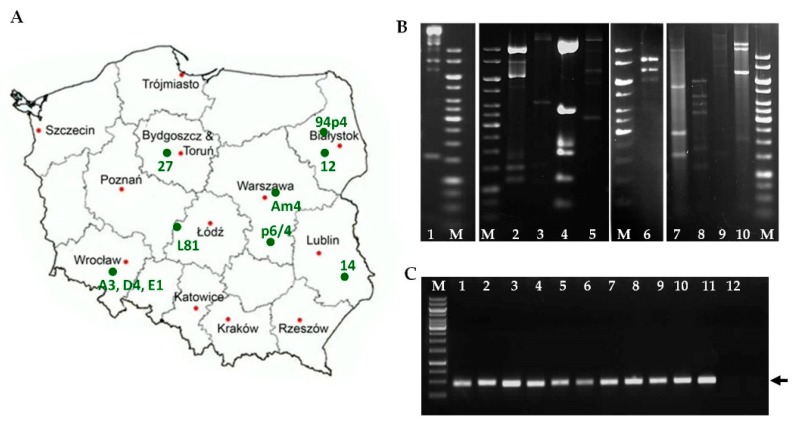
Geographical distribution, restriction fragments length polymorphism (RFLP) and PCR analysis of *Ceduovirus* phages. (**A**) Map shows the geographical site of isolation of each individual phage. (**B**) Restriction fragments length polymorphism (RFLP) analysis of *Ceduovirus* phages using the *EcoRV* enzyme. DNA samples were digested o/n at 37 °C with *EcoRV*. To dissociate *cos* ends prior to electrophoresis 50% formamide was added. M: 1-kb DNA Ladder (Fermentas). Unique phage DNA restriction patterns are as follows: 1, L81; 2, 14; 3, 27; 4, 94p4; 5, p6/4; 6, 12; 7, A3; 8, Am4; 9, D4; 10, E1. (**C**) PCR-based typing of *Lactococcus* phages analyzed in this study. Unique fragments of phage genomes were amplified using primers complementary to the conserved genomic region of *Ceduovirus* phages (*mcp* gene). The arrow indicates the position of a 474-bp band specific to the *Ceduovirus* group. M: 1-kb DNA Ladder (Fermentas), lanes 1–10: DNA fragments generated from respective phage lysates which served as templates: 14, 27, 94p4, L81, p6/4, 12, E1, D4, Am4, and A3, lane 11: DNA fragment generated from c2 (*Ceduovirus* phage; positive control), lane 12: DNA fragment generated from sk1 (*Skunavirus* phage; negative control).

The phages under examination were derived from whey samples collected over a period of 4 years from disturbed fermentation processes in eight dairy plants across Poland (Figure 1A) [33,48]. For the study, we selected all phages previously classified as *Ceduoviruses*. DNA samples from these phages were subjected to restriction fragments length polymorphism (RFLP) analysis (Figure 1B). Based on the DNA restriction patterns, we distinguished and analyzed 10 distinct *Ceduovirus* phages. By PCR analysis using specific primers, we re-confirmed that all the analyzed phages were *Ceduoviruses* (Figure 1C).

**Figure 2:**
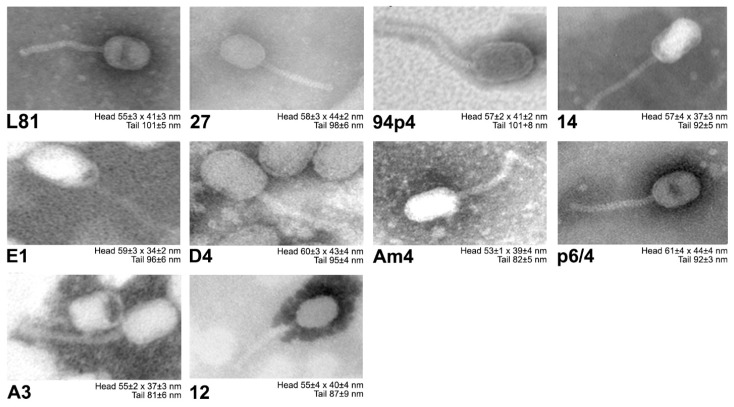
Visualization of the *Lactococcus Ceduovirus* phages using electron microscopy. Head diameter and tail size ± SD given under each electron micrograph were calculated using measure IT 5.0 (Olympus Soft Imaging Solutions GmbH) software as a mean from *n* = 5–10 phages.

Electron microscopy imaging revealed that all of the studied phage isolates possess prolate heads (morphotype B2) and long non-contractile tails, both of which are distinctive for *Siphoviridae Ceduovirus* phages (Figure 2).

### 3.2. Phage-Host Interactions

**Figure 3:**
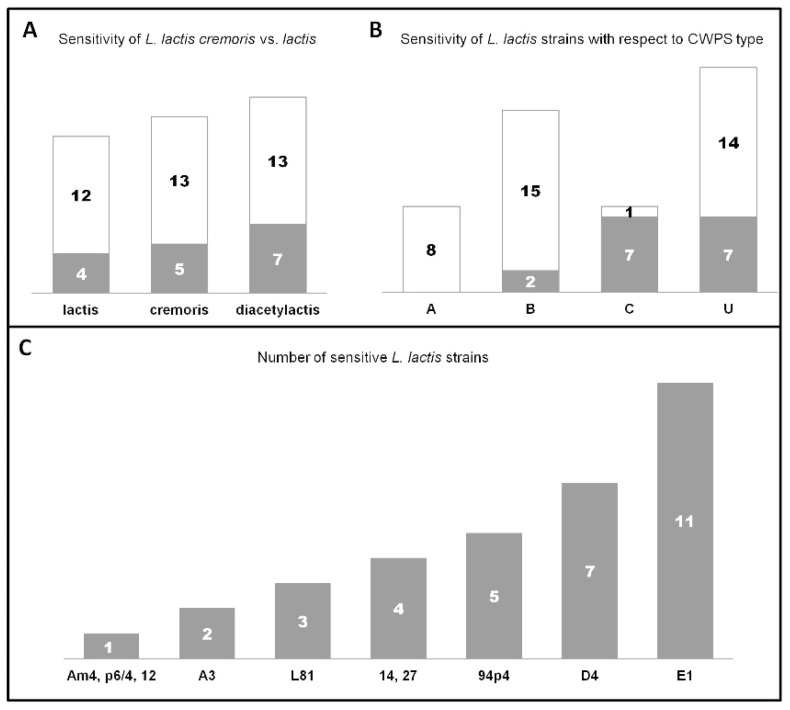
Infectivity of *Ceduovirus* phages towards a set of industrial and environmental *L. lactis* strains. Sensitive strains are marked in gray, resistant strains in white. (**A**) Sensitivity of *L. lactis cremoris* vs. *lactis* subspecies to infection by the analyzed phages. (**B**) Sensitivity of *L. lactis* strains with respect to their cell wall polysaccharide (CWPS) type. Clear lytic zones obtained on double-layer plates containing o/n cultures of a specific *L. lactis* strain (200 µL) and phage lysates (100 µL) were indicative of bacterial sensitivity. (**C**) Number of *L. lactis* strains (from 54 tested) sensitive to infection by individual phages.

The capacity to lyse *L. lactis* cells was tested against a total of 54 isolates deriving from different environments (industrial and natural), and represented by a similar number of subsp. *lactis* (29.7%), subsp. *lactis* biovar. diacetylactis (37%), and subsp. *cremoris* (33.3%) strains (Supplementary Materials Table S1A). As a result of this assay, host infection ranges could be determined. The examined phages were found to possess distinct infection patterns, by lysing different strains. The sole exceptions were phages 14 and 27, for which identical lytic profiles were determined. Almost 30% of all *L. lactis* strains tested were susceptible to phage infection. Among these phage-sensitive strains, 75% of were lysed by two or more phages. Notably, two reference phages, c2 and bIL67, did not form plaques on any of the industrial or environmental strains tested. Moreover, results of this assay revealed that taking together all *L. lactis* subsp. *lactis* strains, they were more frequently lysed by the studied *Ceduovirus* phages (biovar. diacetylactis: 35%; subsp. *lactis*: 25%) than the *cremoris* strains (27.7%) (Figure 3A).

Five out of ten phages were determined to present a relatively broad host range lysing four or more strains (Figure 3C). In this aspect, phage E1 was the most exceptional, infecting a total of 11 strains. Only three phages (Am4, p6/4, and 12) exhibited a restricted host profile limited to one *L. lactis* strain. The phage infection pattern was additionally tested on three laboratory *L. lactis* strains: IL1403, MG1363 and NZ9000 (Supplementary Materials Table S1B). In this assay, a clear subspecies preference was observed. Phages 14, 27 and Am4, similarly to the reference phage bIL67, exhibited lytic activity only on the IL1403 (biovar. diacetylactis) strain. In contrast, phages A3, L81, D4, and 12 formed clear plaques on the *cremoris* strains MG1363 and NZ9000, like the reference phage c2. The three remaining phages, 94p4, E1, and p6/4, were most particular, as they poorly lysed the IL1403 strain (decreased titer compared to their reference strains) (Supplementary Materials Table S1B). Additionally, E1 and p6/4 could not develop on either MG1363 or NZ9000. Such subspecies correlation with the lytic spectrum was less evident for industrial and environmental *L. lactis* strains. Only phages 14 and 27 exhibited an explicit preference for biovar. diacetylactis, lysing four out of 20 strains (20%) from this subgroup, whereas the three narrow host range phages propagated on single strains: *cremoris* (phages 12), *lactis* (phage Am4), and biovar. diacetylactis (phage p6/4). In turn, phages with the widest host profile (94p4, D4, and E1) infected *L. lactis* strains from both subspecies.

Among phages with the widest host range (lysis of > 4 *L. lactis* strains), phages E1 and D4 were the only ones that infected bacteria deriving from natural environmental samples. These two phages presented also a similar lytic spectrum against *cremoris* and *lactis* strains, excluding biovar. diacetylactis (Supplementary Materials Table S1A). Moreover, phage E1 was the only isolate exhibiting an overlapping host range pattern with phages A3, D4, and L81 (group 1) and 14, 27, and 94p4 (group 2).

In previous works, cell wall polysaccharides (CWPS) of bacterial hosts were determined to be a crucial factor in the initial phase of phage adsorption [49,50]. Currently three CWPS types are recognized: A, B, and C, and group U of so-far unknown type(s) [19,32]. In order to elucidate the influence of this factor on phage adsorption, we analyzed the infection patterns with regards to the CWPS type of *L. lactis* cells. Among all industrial and environmental strains tested 31.5% possessed the B type CWPS, while C and A types were equally present in 14.8% of the cells, and 38.9% had an uncharacterized (U) CWPS genotype.

Our host infection study showed that among susceptible industrial strains (16 out of 51) an equal percentage (43.75%) had either the C or U type CWPS, while only 12.5% of phage-sensitive strains had a confirmed B-type genotype. None of the phages lysed A-type CWPS *L. lactis* strains (Figure 3B). When taking into account the whole set of *L. lactis* strains tested, four out of the ten studied phages infected only *L. lactis* strains of a specific type CWPS—phages A3 and 12 lysed only cells with type C CWPS, and phages Am4 and p6/4 with type B CWPS. The remaining six phages formed plaques on *L. lactis* strains with C and U, B and U, or B, C and U CWPS genotypes (Supplementary Materials Table S3).

### 3.3. Infection Dynamics in One-Step Growth Tests

The results of one-step growth assays determined for phages examined in this study a latent period of 19 ± 2 to 28 ± 0 min, and burst time of 27 ± 2 to 33 ± 0 min (Table 3). Despite the rather uniform latent periods and burst times, the burst size of the phages varied and ranged from 32 ± 3 to 197 ± 17 particles per cell. Phage lytic activity was also studied by examining the morphology and plaque size, which ranged between 2 and 5.3 mm. Additionally, for some phages, turbid halos around the plaques were detected. Such plaque morphology may be indicative of phage-encoded depolymerase activity on bacterial cell wall exopolysaccharides.

### 3.4. Phage Genome Sequencing and Comparative Analysis

Whole-genome sequencing of 10 *Ceduovirus* phages isolated from industrial whey samples revealed their genetic content and allowed comparison to other phages from this group. Genome sizes of the characterized phages ranged between 21,458–22,703 base pairs, with an average G-C content of 36% (Table 4). Phage genomes were determined to be organized into two oppositely-oriented blocks, corresponding to the early and late transcribed genes. These two regions were established to contain 18–21 early and 18 late open reading frames (*orfs*).

**Figure 4:**
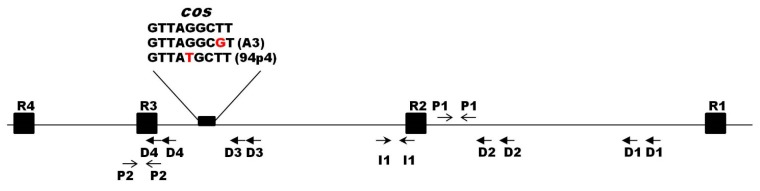
Schematic representation of the cos region of the sequenced *Ceduovirus* phages. A small black rectangle marks the position of the cos site, and its 9-nt sequence is given above (differences in nucleotides are marked in red); black squares (R) mark putative terminase binding sites; arrows depict palindromic sequences (P), direct (D), and indirect (I) repeats.

At the extreme regions of the analyzed phage genomes, 9-nt cohesive ends (cos) were recognized. For eight out of ten phages, the sequence of the cos sites was identical (5′-GTTAGCTT-3′) and matched that of phage c2. The cos sites of phages A3 and 94p4 differed by a single nucleotide each (Supplementary Materials Figure S1). Other features typical for the region surrounding the *Ceduovirus* cos sites were also identified and included four direct (D1–D4) and one inverted (I1) repeats, as well as two palindromic sequences (P1–P2) (Figure 4). Moreover, four 19-bp putative terminase binding sites (R1–R4) were recognized based on analogous sites previously reported for phage c2 [51].

**Figure 5:**
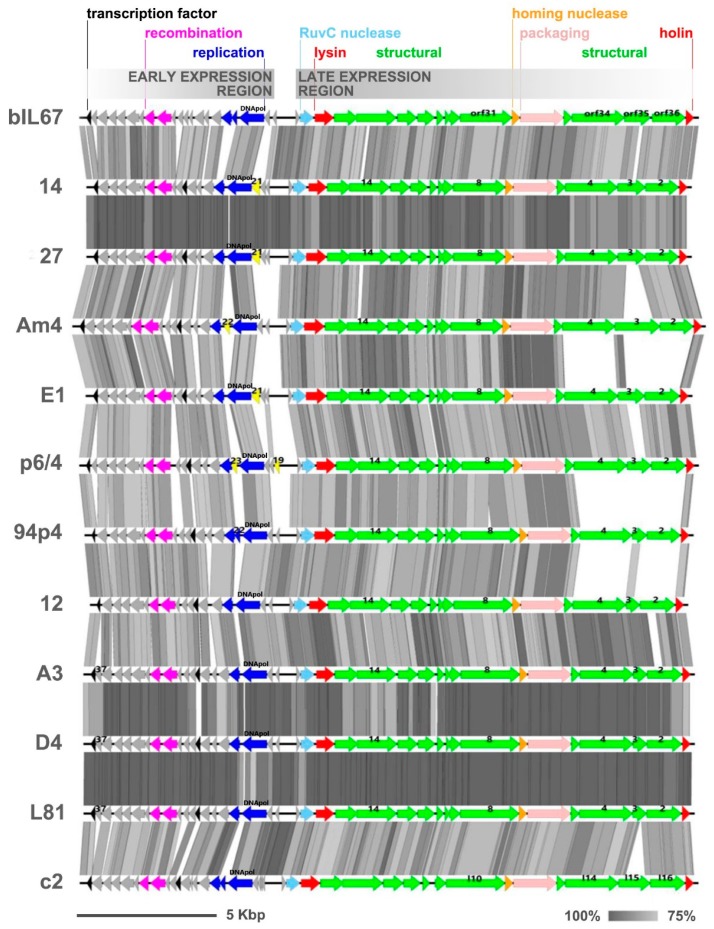
Synteny analysis of complete genomes of *Lactococcus Ceduovirus* phages sequenced in this study. The two transcriptional regions are oriented divergently to each other and are marked above the scheme. Arrows represent open reading frames (*orfs*); gray arrows indicate *orfs* of unknown function, yellow indicates unique *orfs* with unknown function, and the remaining colours indicate *orfs* with annotated functions. The genomes were aligned with respect to the position of the *cos* sites. Each genome is compared with the succeeding genome. The genomes of phages c2 and bIL67 are shown for reference. The gray shading between genomes indicates regions of similarity based on tblastx comparison. Numbers above individual *orfs* are given as reference to the genes discussed in the text.

The genomes of the sequenced phages exhibited highly similar gene synteny, resembling that reported for two reference *Ceduovirus* phages, namely bIL67 and c2 [7,8] (Figure 5).

**Figure 6:**
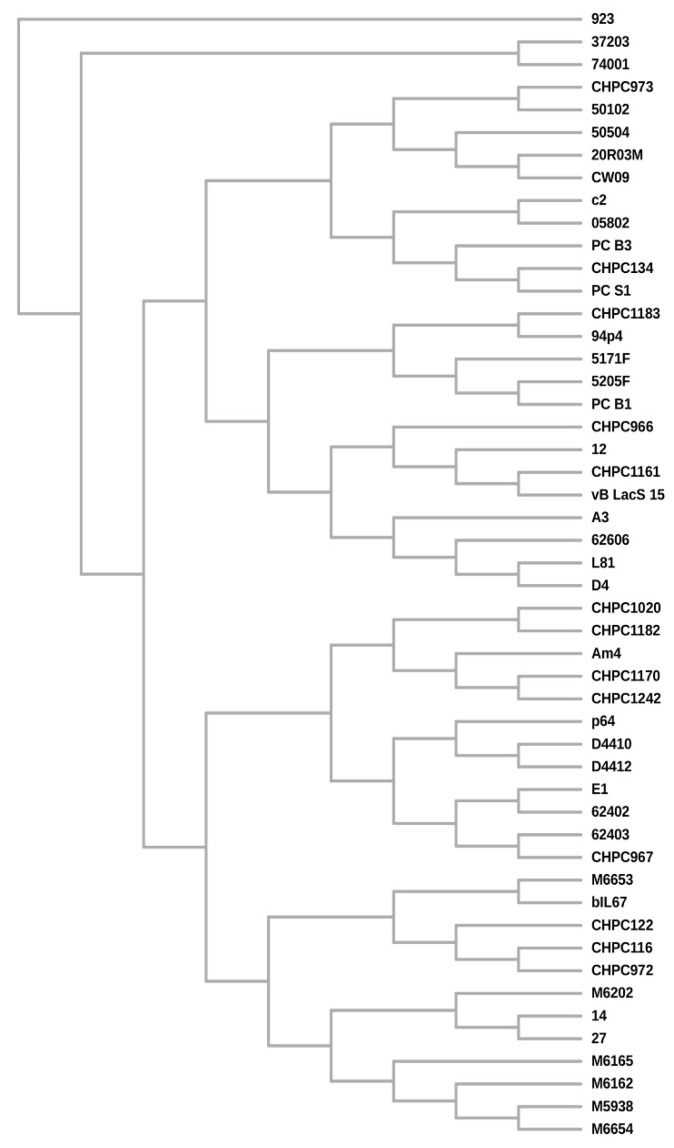
Cladogram of the *Ceduovirus* phages based on the reconciled trees for the following proteins: holin, lysin, ERF-like recombinase, DNApol, tail fiber (GP2 and GP4), major capsid (GP14).

BLAST pairwise alignments with a set of whole phage genomes revealed an overall 86%–99% of nucleotide identity level between the individual phages (coverage 75%–100%). Average nucleotide identity (ANI) calculations confirmed over 80% of identity among the Polish phage isolates (Supplementary Materials Figure S2). Phages 27 and 14 (group A) and D4, A3, and L81 (group B) each shared more than 95% ANI, suggesting the highest similarity among these phages. Interestingly, while phages D4 and A3 from group B derived from the same dairy plant, L81 was isolated from a distant location. Also, group A phages were isolated from distinct geographical sites. An ANI level above 80% was also determined between the analyzed phages and other *Ceduoviruses* present in the GenBank database. In this case as well the nucleotide identity was often higher for phages from distant locations than for indigenous isolates; for instance phage 12 shares >95% ANI with phage CHPC1161. These observations were confirmed by constructing a supertree (Figure 6) based on separate multiple alignments of selected gene products (DNApol, Essential Recombination Function (ERF)-like recombinase, holin, lysin, GP2, GP4, and GP14) and homologous proteins of other *Ceduovirus* phages accessible in the GenBank database (ML trees for each multiple sequence alignments are shown in Supplementary Materials Figure S3A–G). The supertree shows separation of the phages into two major subgroups. The newly sequenced phages are present in both subgroups and show close clustering with other *Ceduovirus* phages from distinct geographical locations (Supplementary Materials Figure S3A–G).

All-against-all pairwise alignments of nucleotide sequences of the early transcribed gene regions revealed 85%–99% of identity (with 76%–100% coverage) among the studied phages. Within this region, two modules encoding putative recombination (ERF-like: pfam 04404 and SSB: pfam 16773) and two to three replication (DNApol) protein products (one with the RecA-like_NTPase superfamily domain cl28885) were annotated by RAST coupled with BLASTp and HHPRED analyses. In spite of the overall similarity, the sequence divergence between phage genomes was observed to result from variations in the length and in the number of identified *orfs* directly upstream of the recombination gene module and the sequence surrounding the putative replication genes (Figure 5). The product of one of these *orfs*, *gp37*, found in phages D4, A3, and L81, was predicted to contain a sterile alpha motif (SAM)-like fold (40.7% confidence, 66% coverage) characteristic for the RuvA domain 2-like superfamily. A truncated version of *gp37*, lacking the last nine nucleotides, was found in phage p6/4. Interestingly, GP37-like proteins with unknown function were detected in only four other *Ceduovirus* phages: bIL67, D4410, D4412, and M6654. Another distinct *orf* was detected in phages 94p4 (*gp22*), p6/4 (*gp23*), and Am4 (*gp22*) between two *orfs* constituting the putative replication module. The *gp22*-encoded product of phage 94p4 exhibited similarity to proteins of unknown function encoded by other *Ceduovirus* phages in the same genomic position. In contrast, the products encoded by the two remaining phages appeared to be largely unique: the p6/4 *gp23* product exhibited no significant hits to any known proteins in the databases, whereas homologs of the Am4 *gp22* product were identified only in phages LW81 and 949, belonging to another *Lactococcus* phage group (949-like). This finding indicates events of horizontal gene transfer (HGT) between different types of lytic lactococcal phages. Another distinct *orf*, *gp21*, was mapped in the early gene region in phages 14, 27, and E1. Its putative product was determined to be a homolog of a hypothetical protein from a *Lactococcus* P335-like phage 98204 (72%–73% identity, 97% coverage) as well as products encoded by various *L. lactis* strains, implying a possible HGT between prophage sequences and lytic phage genomes. Notably, the *gp21* product was found to possess an EF-Ts (elongation factor Ts) domain-like fold. Alignment studies using ClustalOmega showed sequence conservation to the C’ terminal part of *Escherichia coli* EF-Ts (GI: 67462330) involved in dimerization and protein–protein interactions (data not shown). Gene products carrying the same fold have been previously detected in distant phages [52]. As EF-Ts catalyze the discharge of GDP from elongation factor Tu (EF-Tu) and facilitates EF-Tu binding to GTP, it was hypothesized that the phage-encoded EF-Ts-like protein may increase the rate of translation, facilitating phage propagation. Finally, a unique *orf*, designated as *gp19*, was detected at the end of the early gene region of phage p6/4 with no hits in BLASTp searches. Other sequence divergences in the early gene region of the studied phages were linked with the presence/lack of *orfs*, which putative products exhibit high sequence similarity to proteins encoded by other *Ceduovirus* phages, but which function remains unknown.

The late transcribed gene region of the studied phages encodes a conserved number of *orfs*. The nucleotide identity inferred from all-against-all comparison by pairwise alignment of this region was 86%–99% (with 69%–100% coverage). Comparative analysis of the late gene cluster revealed lower sequence conservation within the *gp2-gp3-gp4* region, exhibiting analogy to *orf36-orf35-orf34* of bIL67 (locus_tag: bIL67_gp02-gp03-gp04) and *l16-l15-l14* of c2 (locus_tag: c2p38-c2p37-c2p36), suggested to encode structural proteins, possibly adhesion fibers, involved in host range determination [8,15]. Further divergences were noted within *orfs* for which potential products were annotated as putative tape measure protein (TMP; GP8) and major capsid protein (MCP; GP14). The deduced amino acid sequences of all products were subjected to careful examination in order to correlate them with the host spectrum.

The *gp2-gp3-gp4* region showed the largest sequence divergence within *gp3* (Figure 5), which resulted from variations both of the length (from 169 AA to 542 AA) and sequence of its putative product. Multiple sequence alignment of the *gp3* product of the phages sequenced in this study, and respective proteins of other *Ceduovirus* phages, revealed high similarity at the N’ terminus and divergent middle and C’ parts (Supplementary Materials Figure S4). Notably, the N’ end of the *gp3* product of phages 27, 14, 94p4, Am4, and E1 exhibited significant sequence conservation (>95% identity) to the first 100 AA of *orf35* product (ORF35_bIL67_) and *l15* product (ORF*l15*_c2_). For the remaining phages, the N’ terminus of the *gp3* product demonstrated lower sequence similarity to ORF35_bIL67_ and ORF*l15*_c2_ (75%–77% identity), and resembled more the hypothetical protein (YP_009278611.1) of phage D4412 (>95% identity). Divergence was also noted in the C’ terminal part of GP3 and could be divided into products homologous to the last 65 AA of the C’ terminus of ORF*l15*_c2_ (>80% identity; phages E1, p6/4, A3, L81, D4, and 12) or 55 AA of ORF35_bIL67_ (>85% identity; phages 27, 14, 94p4, and Am4). In turn, BLASTp analyses of *gp3* products longer than 300 AA showed that they are most divergent in the central part, but overall exhibit the highest similarity to ORF35_bIL67_ (59%–62% identity; 98%–100% coverage). The putative products of *gp2* and *gp4* showed minor variations in length and AA sequence, which included short deletions and/or substitutions. Based on the calculated size of GP4, phages in this study could be divided into those encoding a 628- (phages 94p4, Am4, 14, and 27) or 638-AA (phages E1, p6/4, 12, A3, D4, and L81) protein. BLASTp all-to-all comparison showed high similarity (>90% identity) of *gp4*-encoded products within each of these two groups, and a generally low intergroup similarity (39%–42% identity). The exceptions were phage 94p4 and p6/4, which both exhibited lower similarity (80% and 88% identity, respectively) to other *gp4* products within their groups. In turn, comparative analysis of the deduced AA sequence of GP2 showed two conserved groups with low intergroup similarity (≤40% identity). In general, each of the two groups possessed a highly conserved N’ (first 60 AA) and C’ (last 200 AA) end, and a more variable central part. The first group comprised *gp2*-encoded products from phages Am4, 14, and 27 (≥90% sequence identity; 397 AA), and from phage 94p4 (72%–74% identity; 401 AA). The second group of GP2 products was more diverse in length (413–418AA) and in sequence (78%–99% identity). Within this group, a highly conserved (99%–100% identity) subclass of GP2 products could be distinguished for phages A3, L81, and D4.

As we believe the *gp2-gp3-gp4* encode for tail fibers involved in adhesion, an attempt was made to correlate the sequencing data of their putative products with the host infection pattern of the studied phages (Supplementary Materials Table S3). Yet, we did not observe any direct link between the AA sequences of these products with the subspecies or the CWPS type of phage-susceptible strains. Notably, phages 14 and 27 infected the same hosts with CWPS of U type, but did not encode identical *gp2-gp3-gp4* products, and, conversely, phages L81 and D4, for which the AA sequences of *gp3* and *gp4* products were 100% identical, propagated on different hosts. However, when results of host range studies were limited to reference laboratory strains, it was observed that phages with the smallest (169 AA) *gp3* product produced clear plaques only on *L. lactis* subsp. *cremoris*, while phages encoding the longer protein version also infected *L. lactis* subsp. *lactis*. Further studies are needed to better understand this observation. Lower sequence conservation was also found within *gp8*, of which the product was annotated as a putative tape measure (TMP) due to the presence of a specific domain (TIGR02675) typical for phage proteins known or predicted to act as the tail tape measure. Dissimilarities were due to both the length and the deduced AA sequence (72%–96% identity) of the *gp8* product. In phages bIL67 and c2, the corresponding products encoded by *orf31* and *l10* were suggested to play (along with *orf34-orf35-orf36* and *l14-l15-l16*, respectively) a role in host range determination [8,15]. Comparative analyses accompanied by BLASTp searches allowed us to distinguish four groups of *gp8*-encoded products based on their high identity (>90%) with proteins of previously sequenced *Ceduovirus* phages: hypothetical protein of phage CHPC1161 (protein id: QGT52480.1)—L81, D4, A3; tail assembly protein of phage CHPC134 (protein id: QGT52852.1)—12, 94p4; phage CHL92 ORF5 (protein id: AAO15644.1)—p6/4; and phage bIL67 ORF31 (protein id: NP_042314.1)—Am4, E1, 27, and 14. The bIL67- and CHL92-like *gp8* products are of the same length (620 AA) and differ by single AA substitutions. The CHPC1161- and CHPC134-like products are longer (706 AA) and carry an 86-AA insertion in the central part of the protein and exhibit high similarity to ORF*l10* of phage c2. Otherwise, the AA sequence of the N’ and C’ terminal part for both groups are largely conserved. For discrimination purposes, in our analyses, we called these two groups: bIL67-like and c2-like. Relatedness of the *gp8*-encoded products was confirmed by phylogenetic analyses using the maximum-likelihood algorithm (Supplementary Materials Figure S5). The ML tree shows a clear separation of *gp8* products into two branches, correlating with their size. Analysis of the deduced AA sequence allowed us to distinguish one full and one to two incomplete motifs (ER-hand calcium-binding domain) known to be implicated in binding Ca^2+^ and required for efficient adsorption of *Ceduovirus* phages to bacterial cells [53]. For phages 14, p6/4, 94p4 and Am4, a YqbO (lysozyme-like) domain was detected in the N terminal part of GP8, which we believe may play role in peptidoglycan-degradation of host cell walls. Remarkably, phages carrying this domain infected only the *lactis* subspecies, except 94p4 which also infected *cremoris* strains. For phages 27 and E1, the YqbO domain was not detected by computational tools, but the sequences were highly identical across the 1–420 AA region spanning the domain (only one AA mismatch). All of these results corroborate the role of GP8 in host recognition/adsorption and entry of phage DNA. Interestingly, a connection between the deduced AA length of GP8 and infection pattern could be observed (Supplementary Materials Table S3). Phages encoding 620-AA GP8 infected only *L. lactis* subsp. *lactis* strains, whereas phages encoding the longer product could propagate also on *L. lactis* subsp. *cremoris* hosts. The sole exception was phage E1, which encoded the shorter version of the putative GP8, but was able to lyse *L. lactis* from both the *cremoris* and *lactis* groups.

Lower sequence conservation was also detected within *gp14*, for which the potential product was annotated as a putative major capsid protein (MCP). Detailed multiple alignment studies allowed us to establish that these differences were rather minor—the overall AA sequence identity of GP14 was >90% (98%–100% coverage). The lowest similarity was observed for GP14 of phages 14 and 27, for which the deduced sequence contained an additional four AA in the central part compared to the other phages tested. Yet, this difference did not correlate with capsid size (Figure 2) or host range of the phages (Supplementary Materials Tables S1 and S3).

### 3.5. Identification and Functional Analysis of the Origin of Phage Replication

The origin of replication has been previously identified in *Ceduovirus* phages in the intergenic region between the two divergently-oriented modules comprising, respectively, the early and late genes [8]. Multiple alignments of the intergenic region from phages studied in this work and the previously identified replication oris of *Ceduovirus* phages allowed us to classify them into specific ori groups: c2-, bIL67-, or 923-like (Supplementary Materials Figure S6).

Overall, the replication origins within each group were highly conserved (>90% sequence identity) and presented no significant intergroup similarity. Among the three recognized ori types, the c2-like ori was most frequently represented and was found in six out of ten sequenced phages, the bIL67 ori type was determined in three, and 923-type in only one phage. For all phages, the analyzed region was found to be flanked by two putative promoter sequences, which are the first two promoters of the early transcribed gene region (P_E_1, P_E_2). The region within these two promoters did not contain any *orfs* ≥ 100 bp. No ribosome-binding sites downstream of the putative P_E_1 were detected. Both of these features imply that the examined DNA fragments do not encode any protein products. To confirm that these regions contain the putative phage origin of replication, we examined their capacity to carry out plasmid replication in a non-replicative vector in *Lactococcus* cells. For this, representatives of each ori type were cloned in the *E. coli* pUN121 vector, lacking a functional origin in *L. lactis* cells. A set of ori^+^ vectors were obtained: pUN121:ori27 (bIL67-ori type), pUN121:orip6/4 (923-ori type), pUN121:ori94p4 (c2-ori type), and pUN121:oric2 (method control). The ability of phage ori regions to support replication was tested by introducing the ori^+^ vectors into *L. lactis* cells and screening for transformants resistant to chloramphenicol. The results showed that all ori-test plasmids were successfully introduced into *L. lactis* IL1403 wt (subsp. *lactis*) and MG1363 (subsp. *cremoris*) (Supplementary Materials Table S2). This observation holds with the notion that the tested putative phage ori fragments are able to support plasmid DNA replication without additional phage functions in both *L. lactis* strains.

## 4. Discussion

Phages belonging to the *Ceduovirus* group are the second most persistent group of lytic *Lactococcus* phages in dairy plants worldwide. Biodiversity studies have shown the dominance of lytic *Ceduovirus* phages in whey samples only in several countries (e.g., Canada, Germany, Russia, Belarus, and the Czech Republic) [10,54,55]. This observation seems intriguing given the fact that *Ceduovirus* phages have been repeatedly described to have a much wider host range compared to *Skunavirus* phages, and it would be expected that they would encounter a permissive strain more frequently and spread in the environment more easily [10,33,56,57].

In order to gain more knowledge concerning *Ceduovirus* phages in the Polish dairy environment, we investigated the physiological features and performed whole-genome sequencing coupled with comparative analysis of 10 lactococcal phage isolates. The phages derived from whey samples collected over a four-year period in eight different geographical locations. The lytic nature of *Ceduovirus* phages was confirmed by observing a rapid lysis of *Lactococcus lactis* cells. The relatively short latent period of the studied phages is comparable to that of other lactococcal phages detected in dairy settings. In general, lactococcal phages exhibit a latent period of 20 to 60 min, and a burst size in the range of 42 to 400 plaques per cell [58]. The determined burst sizes (32 ± 3 to 197 ± 17 plaques per cell) and high titers (>1 × 10^9^ PFU mL^−1^) on reference starter strains indicate effective phage propagation and an overall good adaptation to the dairy environment. This observation is further supported by the fact that two reference lactococcal phages, bIL67 and c2, were unable to lyse any of the industrial strains tested. It may also imply the ability of industrial phages to bypass potential bacterial phage-resistance mechanisms; however, this notion would need further investigation. A majority of the tested phages were found to infect from 4–11 *Lactococcus* strains of different origin (industrial, environmental, laboratory), which corresponds to a relatively wide host range. Among them, phages D4 and E1 presented the widest lytic spectrum. They were the only ones that could lyse *L. lactis* cells from natural sources, indicating a particular adaptation of these phages to both dairy and natural environments. The presence of a turbid halo around the plaques of D4 and several other phages may signify production of carbohydrate-degrading enzymes. This property is regarded as a competitive/adaptive advantage that increases the fitness of the phage to certain niches [59].

The preference of the studied phages to lyse either *cremoris* or *lactis* (including biovar. diacetylactis) strains was observed, and was especially clear when analyzing laboratory strains but less apparent when considering the whole subset of *L. lactis* strains. This discrepancy may possibly be due to phage-resistance mechanisms (e.g., plasmid- or prophage-encoded) carried by the starter bacteria. Overall, the highest percentage of *L. lactis* phage-sensitive strains belonged to biovar. diacetylactis. A similar preference for biovar. diacetylactis strains has been reported previously also for Polish *Skunaviruses* [31]. Such results may indicate the dominant use of biovar. diacetylactis starter strains in Polish dairy factories and, subsequently, adaptation of phages to this particular host type. Remarkably, not all phages were efficient in lysing the model, laboratory bacteria (IL1403, MG1363, and NZ9000). Phages E1, and p6/4 exhibited poor lysis (turbid, small plaques, low titer compared to their reference strains) of the IL1403 strain, while the *cremoris* type strains (MG1363 and NZ9000) were fully resistant to these phages. Recently, it has been shown that resident prophages may influence host susceptibility and the phage plaquing efficiency [60]. This effect may be due to a yet undermined prophage-encoded anti-*Ceduovirus* defense mechanism, similar to the described super-infection exclusion (Sie) phenomenon against *Skunavirus* phages. Thus, the potential factors influencing the lytic pattern of the analyzed phages, including the prophage effect, need further exploration.

The sequenced phages show a preference for strains with type C CWPS, which is in line with previous reports on *Ceduoviruses* [10]. Based on this analysis, they were grouped into two types (i) phages infecting hosts with a specific CWPS type (B—for phages Am4 and p6/4, and C—for phages A3 and 12) and (ii) those which lyse hosts with different saccharidic receptors (six remaining phages). We may speculate that the first group of phages recognizes and adsorbs to unique components of specific CWPS types, while phages from the second group interact with a common CWPS determinant. Additionally, all strains with type A CWPS tested in this study were phage-resistant. Given these results, it seems that the bacterial cell wall components may be a crucial parameter that should be taken into account when selecting for phage-resistant industrial starter strains.

All versus all genome comparisons revealed more than 80% average nucleotide identity between the studied phages and other *Ceduoviruses* found in GenBank. Several phages shared >95% ANI, a value which is generally regarded as a cut-off for species discrimination. Remarkably, no general correlation with their geographical origin could be found; often phages isolated from different settings exhibited high identity, including as examples phages 12 and CHPC1161 or 14 and 27. Phylogenetic studies showed separation of *Ceduoviruses* into two major subgroups and confirmed the relatedness of phages from distant locations. In the case of phages 14 and 27, their high similarity was also reflected in the results of our host range and one-step growth studies. It could be that these two phages originated from the same parental phage, which was carried over by humans or as a contamination in starter media to different dairy plants where it diverged. Assessment of the link between phage phylogeny and the site of their isolation may be also impaired by the global distribution of common industrial starters. Only further analysis could provide informative data concerning the occurrence and evolution of related phages in geographically separate regions.

Apart from host factors, results of our analyses suggest that the *gp8* product of the studied phages may be correlated with their lytic spectrum. GP8 has been annotated as a putative tape measure based on the presence of TIGR02785 domain typical for phage TMPs. In other phages, tape measure proteins have been implicated in several crucial steps of phage infection, including tail length determination and entry of phage DNA into the host cell. The ORF*l10* of c2 and ORF31 of bIL67, encoded by genes located in the same genetic context, were suggested to play a role in host range determination [8,15]. Based on the similarity of GP8 to these two products, two (c2-like and bIL67-like) groups varying in the length of AA sequence were detected. Remarkably, phages encoding longer GP8 (c2-like) were capable of lysing *cremoris* and, in some cases, *lactis* strains of C or U CWPS type. Shorter products (bIL67-like) were restricted to phages infecting solely subsp. *lactis* cells of B or U CWPS type. The exceptions were phages 94p4 and E1, which both formed plaques on *lactis* and *cremoris* strains (B, C, or U type), but encoded for GP8 of a different length. These observations allow us to assume that the recognition of particular lactococci hosts with a specific CWPS type is dependent on the AA composition/length of the GP8. Yet, the exact function of GP8 remains obscure. In general, phage TMPs are acknowledged to play role in DNA injection by forming a channel that reaches through the thick peptidoglycan layer, typical for Gram-positive lactococci, into the host cell. The presence of a Ca^2+^ binding motif and peptidoglycan-degrading (YqbO) domain (for shorter products) within the GP8 support the notion of its function as a tape measure protein. Further data will be needed to resolve this issue. Generating a series of hybrid *gp8* products, differing in length and presence of specific motifs and domains, may provide more data on the crucial sequence elements that correlate with host range.

As suggested by previous studies on c2 and bIL67 model *Ceduovirus* phages, product(s) of the *l16-l15-l14* and *orf36-orf35-orf34* gene clusters, respectively, might encode for another host range determinant(s) [15]. Our comparative analysis of analogous phage products encoded by *gp2-gp3-gp4* did not reveal any correlation of AA sequence divergence with host range. Within the *gp2*-*gp3*-*gp4* region, the *gp3* product exhibits the largest variation in length and might have the biggest impact on the phage host range. However, this assumption would need further examination, as phage Am4 encoding the longest (542 AA) putative *gp3* product identified so far in *Ceduovirus* phages lysed only 1 out of 54 examined *L. lactis* strains. The widest spectrum of infection was noted for phage E1, of which the *gp3* product was most similar in AA sequence to that of phage 27, able to effectively lyse only four strains. Thus, it seems that there are other proteins (or factors) engaged in host range determination. Notably, the lytic spectrum of phage E1 overlapped with that of phages infecting exclusively *cremoris* or *lactis*/diacetylactis strains. Thus, it may be that phage E1 is a hybrid between c2-like and bIL67-like phages. Yet, we cannot exclude that the broad host range of E1 is rather the effect of phage evolution due to starter strain rotation—a common routine applied in dairy plants to limit phage infections. Such an approach in the long term is suggested to favor the appearance of phages with an extended host range [18].

Sequence analysis of the putative *ori* region confirmed the presence of three distinct *ori* types, with c2 *ori* type the most frequently observed. This is in line with previous studies on a much larger set of phages from New Zealand where the c2 *ori* type also prevailed [14]. To date, no recombinant *oris* have been detected, which strongly implies a general sequence conservation of the three *ori* types, irrespective of geographical location. Our studies prove that the identified *ori* regions serve the same function and support plasmid replication without any additional phage proteins. Moreover, we show that the *ori* regions are functional in both *lactis* and *cremoris* strains, regardless of the lytic spectrum of the phage itself. This suggests that other host-phage factors than *ori* incompatibility influence the observed host range.

## 5. Conclusions

The role of *Ceduovirus* phages in shaping dairy environments should not be underestimated. With our study, we anticipated to deepen our knowledge on host-phage interactions and genetic diversity in relation to already sequenced *Ceduovirus* phages. The results of our study suggest a link between the putative tape measure protein and host range. Phylogenetic studies and average nucleotide identity calculations have shown that highly similar phages can occur in distant geographical locations. To our knowledge, this is the first reported research on *Ceduovirus* phages that have emerged from dairy plants in Poland. Data on *Ceduovirus* phage characteristics, including preference for specific *L. lactis* hosts and the presence of unique features, provides valuable information that may be used to develop efficient methods of eliminating them from dairy plants and, at the same time, creates a basis for future phage studies.

## Figures and Tables

**Table 1:** Bacterial strains, plasmids, and bacteriophages used in study.

**Bacterial Strains**	**Relevant Features**	**Source/Reference**
IL1403	*L. lactis* subsp. *lactis* wild-type strain	[26]
MG1363	*L. lactis* subsp. *cremoris* wild-type strain	[27]
NZ9000	*L. lactis* subsp. *cremoris* MG1363 derivative *pepN::nisRnisK* strain	MoBiTec^®^
ER2655	*E. coli* strain *F- λ- fhuA2* [*lon*] *ompT lacZ::T7p07 gal sulA11* Δ(*mcrC-mrr*)*114::IS10 R*(*mcr-73::miniTn10-TetS*)*2 R(zgb-210::Tn10*)(*TetS*) *endA1* [*dcm*]	NEB^®^
IBB338, 339, 342, 695-700, 704, 732-739, 741-758, 760-764, 1219, 1267, 1280, 1283, 1298, 1784, 1787-1792, 1796	*L. lactis* strains	IBB PAS collection
**Plasmids**	**Relevant Features**	**Source/Reference**
pG+host3	*E. coli* vector, Ts derivative of pGK12, cat^R^	[28]
pUN121	*E. coli* vector, tet^R^, amp^R^ non-replicative in *L. lactis* cells	[29]
pUN121:catR:ori27	recombinant plasmid with putative ori from *L. lactis* phage 27, cat^R^	this study
pUN121:catR:ori94p4	recombinant plasmid with putative ori from *L. lactis* phage 94p4, cat^R^	this study
pUN121:catR:orip6/4	recombinant plasmid with putative ori from *L. lactis* phage p6/4, cat^R^	this study
pUN121:catR:oric2	recombinant plasmid with putative ori from *L. lactis* phage c2, cat^R^	this study
**Bacteriophages**	**GenBank Accession Number** *	**Source/Reference**
bIL67	NC_001629.1	[7]
c2	NC_001706.1	[8]
bIBB_L81 (L81)	MH779526 *	this study
bIBB_12 (12)	MH779518 *	“
bIBB_14 (14)	MH779519 *	“
bIBB_94p4 (94p4)	MH779521 *	“
bIBB_27 (27)	MH779520 *	“
bIBB_p6/4 (p6/4)	MH779527 *	“
bIBB_A3 (A3)	MH779522 *	“
bIBB_D4 (D4)	MH779524 *	“
bIBB_Am4 (Am4)	MH779523 *	“
bIBB_E1 (E1)	MH779525 *	“

* Complete whole-genome sequences of phages analyzed in this study were deposited in GenBank.

**Table 2:** Primers used in study.

Name	5′-3′ Sequence *
c-2for	CAGGTGTAAAAGTTCGAGAACT
c-2rev	CAGATAATGCACCTGAATCA
ori27_F	CCGGAATTCCAAGCTATCAAATATTTC
ori27_R	CCCAAGCTTCATACCAACAAAAG
orip6/4_F	CCCGATATCCAATGTGTTTTTGTG
orip6/4_R	CCCAAGCTTCCTACAAAAAATTTAG
ori94p4_F	CCGGAATTCTARTTACYTTGCTAAAGGG
ori94p4_R	CCCAAGCTTTCCCTCWTTGATTATG
oric2_F	CCGGAATTCTARTTACYTTGCTAAAGGG
oric2_R	CCCAAGCTTTCCCTCWTTGATTATG
catR_F	CGCGGATCCATGAAGAAAGCAGACAAGTAAG
catR_R	ACGCGTCGACGTAAAAAGTACAGTC
pUN121_F	GTCTGGCTATGCAGAAATCC
pUN121_R	GCTTATAACGCCGCATTGCT
pUN121_catR_F	TATCGACTACGCGATCATGG
pUN121_catR_R	GCGTGCAAGATTCCGAATAC
CWPS_Afor	GTGCCTATGCTCCGTTAGTC
CWPS_Arev	CGAGGGCCAATCTCTTTACC
CWPS_Bfor	GATTCAGTTGCACGGCCG
CWPS_Brev	AGTAAGGGGGCGGATTGTG
CWPS_Cfor	AAAGCTCATCTTTCCCCTGTTGT
CWPS_Crev	GCACCATAGTCTGGAATAAGACC
CTL_for (U-type)	GTACACTATGTTTATAACAATCATCCAG
CTL_rev (U-type)	GCAAACCAGATTCAAAGTCAGTATG

* underlined are restriction sites added to the primer sequence.

**Table 3:** Phage infection parameters calculated from one-step growth curves.

Phage	Burst Size * [Plaques Per Infected Cell]	Latent Period * [min]	Burst Time * [min]	Plaque Diameter [mm]	Halo [mm]
14	66 ± 3	20 ± 0	32 ± 1	2.5	0.5
27	67 ± 2	20 ± 0	29 ± 3	2	0.5
94p4	64 ± 3	28 ± 0	31 ± 0	2	nd
L81	32 ± 3	20 ± 0	27 ± 2	2	1
p6/4	34 ± 3	22 ± 3	29 ± 1	1.5	nd
12	197 ± 17	22 ± 3	29 ± 2	3	nd
E1	109 ± 8	26 ± 2	33 ± 0	5.3	nd
D4	94 ± 4	19 ± 2	28 ± 3	5	1
Am4	33 ± 3	20 ± 0	29 ± 2	3.5	0.5
A3	91 ± 3	23 ± 3	33 ± 0	4	0.5

* Results are a means of at least three experiments ± SD; nd—none determined.

**Table 4:** Characteristics of phage genomes based on sequencing data.

Phage (GenBank Accession No.)	Propagation Strain (Phage Titer; PFU mL^−^^1^)	Genome Length (bp)	GC Content (%)	*Orf* Count
**14** (MH779519)	738 (2.4 × 10^10^)	21,710	36	37
**27** (MH779520)	738 (8 × 10^8^)	21,713	36.1	37
**94p4** (MH779521)	753 (1.1 × 10^10^)	21,834	36.1	38
**L81** (MH779526)	MG1363 (8.4 × 10^9^)	21,915	36.0	38
**p6/4** (MH779527)	739 (1.3 × 10^9^)	22,220	36.0	39
**12** (MH779418)	1787 (6.1 × 10^9^)	21,458	36.2	36
**E1** (MH779525)	1283 (1.3 × 10^10^)	21,749	36.1	37
**D4** (MH779524)	1280 (1.4 × 10^10^)	21,907	36.0	38
**Am4** (MH779523)	1219 (2.4 × 10^9^)	22,703	35.9	38
**A3** (MH779522)	1267 (7.2 × 10^9^)	21,906	36.2	39

## Supplementary Materials

The following are available online at https://www.mdpi.com/1999-4915/12/3/280/s1.
