# Supplementary material for: LactococcusCeduovirus Phages Isolated from Industrial Dairy Plants—From Physiological to Genomic Analyses
Source: Viruses. 2020 Mar 3;12(3):280. doi: 10.3390/v12030280 (PMC7150918; doi:10.3390/v12030280)
Supplement: Supplementary file 1 [file viruses-12-00280-s001.pdf]

Supplementary Figure S1. Organization of the cos region of the sequenced *Ceduovirus* phages. Multiple sequence alignment with the cos region of reference phages c2 and bIL67 was performed using the ClustalOmega tool [1]. Identified cos sequences are marked in gray; putative terminase binding sites (R) are boxed; direct (D) and indirect (I) repeats are marked by whole arrows; palindromic sequences (P) are shown by dashed arrows.

```
                         R4
p6/4     ACAAGAA---ATAATAAAATAG-AAAGTGAGGTCATTACTCTTCAATTACGTGCCACTCAA   505
A3       GCAAAAT---TTAATTAAATAG-AAAGCGAGGTCATTGCTCTTCATTTACACGCCGCTCAA   618
L81      ACAAAAT---TTAATTAAATAG-AAAGTGAGGTCATTACTCTTCATTTACACGCCACTCTT   623
D4       ACAAAAT---TTAATTAAATAG-AAAGTGAGGTCATTACTCTTCATTTACACGCCACTCTT   555
Am4      GCAAAAT---TTAATTAAATAG-AAAGCGAGGTCATAACTCTTCAATTACACGCCACTCAA   677
14       ACAAAAT---TTGATTAAATAG-AAAGTGAGGTCATAACTCTTCAATTACACGCCACCCAA   445
27       ACAAAAT---TTGATTAAATAG-AAAGTGAGGTCATAACTCTTCAATTACACGCCACCCAA   625
C2       ACAAAAT---TTAATAAAATAGAAAAGTGAGGTATCTCTTCATTTACACGCCACTCAA---   684
bIL67    ACAAAATTATTTATTAAAATAG-AAAGTGAGGTCATAACTCTTCAATTACATGCCACTCAA   811
94p4     ACAAAAT---TTAATAAAATAG-AAAGTGAGGTCATTACTCTTCATTTACACGCCACTCAA   685
12       ACAAAAT---TTAATTAAATAG-AAAGCGAGGTCATTACTCTTCATTTACACGCCACTCAA   565
E1       ACAAAAT---TTGATTAAATAG-AAAGTGAGGTCATAACTATTCAATTACACGCCACCCAA   617

p6/4     AA--GAGTGGTTTTTTTGTTTGGTTGATGATTAGATACCCTTTGCTATATAATACCCCTG   563
A3       ACGAG---TGGTTTTTTGTTTGGTTGTTGACGAGGTATGCCCCACTATATAATGCCCCTG   675
L81      AATTGAGTGGTTTTTTTGTTTGGTTGTTGATGAGGTACGCCCTACTATATAATACCCCTG   683
D4       AATTGAGTGGTTTTTTTGTTTGGTTGTTGATGAGGTACGCCCTACTATATAATACCCCTG   615
Am4      ACGAGTG--GTTTTTTTGTTTGGTTGTTGACGAGGTATGCCCCACTATATAATGCCCCTG   735
14       ACGAGTG--GTTTTTTTGTTTGGTTGTTGATTAGATACCCCTTGCTATATAATACCCCTG   503
27       ACGAGTG--GTTTTTTTGTTTGGTTGTTGATTAGATACCCCTTGCTATATAATACCCCTG   683
C2       TCAAGAGTGGTTTTTTTGTTTGGTTGTTGATGAAGTACGCCCTGCTATATAATACCCCTT   744
bIL67    TCAAGAGT-GGTTTTTTGTTTGGTTGATGATTAGATACCCCTTGCTATATAATACCCCTG   870
94p4     ACGAGTG--GTTTTTTTATTTGGTTGTTGACGAGGTACGCCCTGCTATATAATACCCCTG   743
12       ACGAGTG--GTTTTTTTGTTTGGTTGTTGATGAAGTACGCCCTACTATATAATACCCCTG   623
E1       ACGAGTG--GTTTTTTTGTTTGGTTGTTGATTAGATACCCCTTGCTATATAATACCCCTG   675

p6/4     TAAGCTCACGGATTGGCTTGTATTGCATTTTAAGTAATTTCTAGGATAATGACAAGGAAC   623
A3       CAAGCTCACAGATTGCCTTGTATTGCATTTCAAATAATTTCTAGGATAATGACAAGGAAC   735
L81      TAAGCTCCTAGATTGGCTTGTATTGCATTTTAGATAATTTCTAGGATAATGACAAGGAAT   743
D4       TAAGCTCCTAGATTGGCTTGTATTGCATTTTAGATAATTTCTAGGATAATGACAAGGAAT   675
Am4      CAAGCTCACAGATTGCCTTGTATTGCATTTTAAATAATTTCTAGGATAATGACAAGGAAC   795
14       TAAGCTCACAGATTGGCTTGTATTGCATTTTAAATAATTTCTAGGATAATGACAAGGAAC   563
27       TAAGCTCACAGATTGGCTTGTATTGCATTTTAAATAATTTCTAGGATAATGACAAGGAAC   743
C2       TAAGCTCACAGATTGGCTTGTATTGCATTTTAAATAATTTCTAGGACAATGACAAGGAAC   804
bIL67    TAAGCTCACAGATTGGCTTGTATTGCATTTTAGATAATTTCTAGGATAATGACAAGGAAC   930
94p4     TAAGCTCATAGATTGGCTTGTATTGCATTTTAGATAATTTCTAGGATAATGACAAGGAAT   803
12       TAAGCTCATAGATTGGCTTGTATTGAATTTTAGATAATTTCTAGGATAATGACAAGGAAC   683
E1       TAAGCTCACAGATTGGCTTGTATTGCATTTTAAATAATTTCTAGGACAATGACAAGGAAC   735

                                                                R3′
p6/4     AGACCAAAACACGCAAAATAGAACGATTTAAGAGGAATTACTGCTTATTTTTTTCAAAAC   683
A3       AGACCGAAACATGCAAAATAAGACGTTTTATGAGTAATTACATCATATTTTTTACAAAAC   795
L81      AGACCTAAACACGCAAAATAAAACGATTTACGAGGAATTACGGCATGTTTTTTTCAAAAC   803
D4       AGACCTAAACACGCAAAATAAAACGATTTACGAGGAATTACGGCATGTTTTTTTCAAAAC   735
Am4      AGACCGAAACATGCAAAATAAGACGTTTTATGAGCAATTACATCATATTTTTTACAAAAC   855
14       AGACTTAAACACGCAAAATAAAACGATTTACGAGGAATTACGGCATATTTTTTTCAAAAC   623
27       AGACCTAAACACGCAAAATAAGACGTTTTATGATAAATTATGATATATTTTTTTCAAAAC   803
C2       AGACCAAAACACGCAAAATAAGACGATTTAGGAAGAATTACGGCATATTTTTTTCAAAAC   864
bIL67    AGACCAAAACACGCAAAATAGAACGATTTAAGAGGAATTACTGCTTATTTTTTTCAAAAC   990
94p4     AGACCAAAACACGCAAAATAAGACGATTTATGAGGAATTACGATATATTTTTTTCAAAAC   863
12       AGACCTAAACACGCAAAATAAAACGATTTACGAGGAGTTACGGCATATTTTTTTCAAAAC   743
E1       AGACTTAAACACGCAAAATAAGACGTTTTATGATAAATTATGATATATTTTTTTCAAAAC   795
                                                       P2 ·······►

           R3″                     cos
p6/4     GAAAAATGAAAAAATAACTCCTAAAGAGTTAGGCTTGATAGAAAACCCACCCCCTTTATA   743
A3       AAAAAATAGAAAAATAGATTCCAAAGAGTTAGGCGTGATAGAGAACCCACCCCCTTTATA   855
```

```
L81     GAAAAACGGAAAAATAGATTCAAAAGAGTTAGGCTTGATAGAAAACCCGCCCCCTTTATA    863
D4      GAAAAACGGAAAAATAGATTCAAAAGAGTTAGGCTTGATAGAAAACCCGCCCCCTTTATA    795
Am4     TAAAAATAGAAAAATATATTCCAAAGAGTTAGGCTTGATAGAGAACCCACCCCCTTTATA    915
14      GAAAAATGGAAAAATAGATTCAAAAGAGTTAGGCTTGATAGAAAACCCACCCCCTTTATA    683
27      GAAAAATAGAAAAATAGATTTCAAAGAGTTAGGCTTGATAGAGAACCCACCCCCTTTATA    863
C2      GAAAAATGGAAAAATAGATTCAAAAGAGTTAGGCTTGATAGAAAACCCACCCCCTTTATA    924
bIL67   GAAAAATGAAAAAATAACTCCTAAAGAGTTAGGCTTGATAGAAAACCCACCCCCTTTATA    1050
94p4    GAAAAATGGAAAAATAGATTCCAAAGAGTTATGCTTGATAGAGAACCCACCCCCTTTATA    923
12      GAAAAACTGAAAAATAGATTCAAAAGAGTTAGGCTTGATAGAAACCCCACCCCCTTCATA    803
E1      GAAAAATAGAAAAATAGATTTCAAAGAGTTAGGCTTGATAGAGAACCCACCCCCTTTATA    855
        <----D4 <----D4                                <----D3
        <......P2

p6/4    TCACACCCCCTTTAATCTCTAACAAAATTTAAAACAAAAAAGCACAACAAAGC----TA    799
A3      TCACACCCCCTTTAAACTCTAACAAAATTTAAAATAAAAAAAGCACAACAAAGC----TA    911
L81     TCACACCCCCTAAGAACTCGGAATAAAAATAAAAAAATAATATATAATAAATAACAAATC    923
D4      TCACACCCCCTAAGAACTCGGAATAAAAATAAAAAAATAATATATAATAAATAACAAATC    855
Am4     TCACCCCCCCTATAAACTTGAACAAAAATTTAAAACAGCACAACAA------AGCTACAA    969
14      TCACACCCCCTTTAATCTCGAACAAAAATTTAAAACAAAAAAAGCACAACAAAGCTACAA    743
27      TCACACCCCCTAAGAACTCGAAGTAAAAAATAAAAAAATAATATATAATAACTAACAAAT    923
C2      TTACACCCCCTAAGAACTCGGAATAAAAATAAAAAAAATAATATATAAATAAATAACAAA    984
bIL67   TCACACCCCCTTTAAACTCAAACAAAAATTTAAAATAA---AAAAAGCACAACAAAGCTA    1107
94p4    TCACACCCCCTTCAAACTTGGAAGAAAAAAATTTAAAATAAAAAAACATAGAAAACGCCA    983
12      TCACCCCCCCTTCAAACCATAACAAAAATTTAAAA---CAAAAAAAGCACAACAAAGTTA    860
E1      TCACACCCCCTTCAAACTCGAACAAAAATTTAAAA---CAAAAAAAGCACAACAAAGCTA    912
           <-----D3

p6/4    CAAAG-TTTATTTTGTAACGATTGCTATGCTAT----ACTATCATACTATATACACTAGT    854
A3      CAAAT-TTATTTTTGTAACGATTGCTATGCTAT----ACTATCATACTATATACACTACT    966
L81     AAAAA--AATTATTAATTAAAATATATTATTAT----ATTATATACATCACTACTACTAT    977
D4      AAAAA-AATTTATTAATTAAAATATATTATTAT----ATTATATACATCACTACTACTAT    910
Am4     AGTT----TATTTTGTAACGATTGCTA---TG-CTATACTATCATACTATATACACTAGT    1021
14      AGTT----TATTTTGTAACGATTGCTA---TGC-TATACTATCATACTATATACACTAGT    795
27      CAAA----AAATTTATTAATTAAAATA---TATTATTATATTATATATATCACTACTACT    976
C2      TCAAAAAAAT--TTATTAATT-----AAAATATATTATTATATTGTATACATCACTACTA    1037
bIL67   CAA-ATTTATTTTTGTAACGTTTGCCATGCTATACTATCATACTATAT----ACACTAGT    1162
94p4    CAAATTTTATTTTCGTAACGTTCGCTATGTCATACATCATACTACTATACTACTACACTA    1043
12      CAAAG-TTTATTTTGTAACGATTGCTATGCTATACTATCATACTATAT----ACACTAGT    915
E1      CAAATTTGTTTTTTGTAACGATTGCTATGCTATACTATCATACTATAT----ACACTAGT    968

                                                         R2'
p6/4    ACTATACTACTACTATACTATTCTTCTTTATCTCCTCCGTCTATTTCTTCTTTCTTATTA    914
A3      ACTATACTACTACTATACTATTCTTCTTTATCTCCTCCCTCTATTTCTTCTTTCTTATTA    1026
L81     ACTACTACACTACTATACTATTCTTCTTTATCGCCTCCGTTTATTTCTTCTTTCTTATTA    1037
D4      ACTACTACACTACTATACTATTCTTCTTTATCGCCTCCGTTTATTTCTTCTTTCTTATTA    970
Am4     ACTATACTACTACTATACTATTCTTCTTTATCTCCTCCCTCTATTTCTTCTTTCTTATTA    1081
14      ACTATACTACTACTATACTATTCTTCTTTATCTCCTCCCTCTATTTCTTCTTTCTTATTG    855
27      ACACTACTACACTACTACTATTCTTCTTTATTTCCTCCGTCTATTTCTTCTTTCTTATTA    1036
c2      CTACTACACTACTACTACTATTCTTCTTTATCTCCTCCGCTTGTTTCTTCTTTCTTTTCA    1097
bIL67   ACTATACTACTACTATACTATTCTTCTTTATCTCCTCCCTCTATTTCTTCTTTCTTATTA    1222
94p4    TGCACTACTATACTATACTATTCTTCTTTATCCCCTCCGTTTATTTCTTCTTTCTTATCA    1103
12      ACTATACTACTACTATACTATTCTTCTTTATCTCCTCCCTCTATTTCTTCTTTCTTATTA    975
E1      ACTATACTACTACTATACTATTCCTTTTTATCTCCTCCCTCTATTTCTTCTTTCTTATTA    1028
                               ---I1--->               <---I1---
        R2"
p6/4    GTTAAGAACTTATACAAGTGACTAACTACTACTGCTAAGTATACAGTTCCAACCAATGAT    974
A3      GTTAAGAACTTATACAGATGACTAACTACTACTGTTAAGTATACAGTTCCAATGAGTGTT    1086
L81     GTTAAGAACTTATACAGATGACTGACAACAACAGCAAGATAAGCAGTTCCAATCAATGTT    1097
D4      GTTAAGAACTTATACAGATGACTGACAACAACAGCAAGATAAGCAGTTCCAATCAATGTT    1030
Am4     GTTAAGAACTTATACAAGTGACTGACTACTACTGCTAAGTATACAGTTCCAGTCAATGAT    1141
14      GTTAAGAACTTATACAGATGACTAACTACTACTGCTAAGTATACAGTTCCAATCAATGAT    915
27      GTCAAGAACTTATACAGATGACTAACTACTACTGCTAAGTATACAGTTCCAACCAATGAC    1096
C2      GTTAAGAACTTATACAGATGACTAACTACTACTGTTAAGTATACAGTTCCAATGAGTGTT    1157
bIL67   GTTAAGAACTTATACAGATGACTAACTACTACTGTTAAGTATACAGTTCCAATTAGTGTT    1282
94p4    GTTAAGAACTTATACAAGTGACTGACAACAACTGCAAGATAAACAGTTCCAATCAATGTT    1163
12      GTTAAGAACTTATACAGATGACTAACTACTACTGTTAAGTATACAGTTCCAATGAGTGTT    1035
E1      GTTAAGAACTTATACAGATGACTAACTACTACTGTTAAGTATACAGTTCCAATTAGTGTT    1088
              ......P1..>                  <..P1..      <----D2
```

```
p6/4     ATTAGTTCATCACTAATCATACCTTTAAAGACTGTCATACCTGCAACGTAAACAGATAGA    1034
A3       AATAGTTCATCACTAATCATACCTTTAAACACAGTCATACCTGCAACATAAACAGATAGG    1146
L81      ATTAGTTCATCACTAATCATTCCTTTAAACACTGTCATACCTGCTACATACACAGACAAT    1157
D4       ATTAGTTCATCACTAATCATTCCTTTAAACACTGTCATACCTGCTACATACACAGACAAT    1090
Am4      ATTAGTTCATCACTAATCATTCCTTTAAAGACTGTCATACCTGCAACATAAACAGATAGG    1201
14       ATTAGTTCATCACTAATCATACCTTTAAACACAGTCATACCTGCAACATAAACAGATAGG    975
27       ATTAGTTCATCACTAATCATACCTTTGAACACAGTCATACCTGCTACATACACAGACAAT    1156
C2       ATTAGTTCATCACTAATCATACCTTTGAACACAGTCATACCTGACACATAAACAGACAAT    1217
bIL67    ATTAGTTCATCACTAATCATACCTTTAAAGACTGTCATACCTGATACATAAACAGATAGG    1342
94p4     ATTAGTTCATCACTAATCATTCCTTTGAACACTGTCATACCTGCTACGTACACAGATAAC    1223
12       AATAGTTCATTACTAATCATACCTTTAAACACTGTCATACCTGCTACATACACAGACAAT    1095
E1       AATAGTTCATCACTAATCATACCTTTAAACACAGTCATACCTGCAACATAAACAGATAGG    1148
            <----D2

                                                                  <--
p6/4     ACAATTAACTCTACTGCCATTCTTTTTCTAACGCTAGGTTTTAGAGTACCTGTGTCAATA    1094
A3       ACAATTAACTCTACTGATATTCTTTTTCTAACGCTAGGTTTTAGAGTACCTGTATCTAAA    1206
L81      ACAATTAATTCTACTGTTATTCTTTTTCTAACGCTAGGTTTTAAAGTACCTGTGTCAATA    1217
D4       ACAATTAATTCTACTGTTATTCTTTTTCTAACGCTAGGTTTTAAAGTACCTGTGTCAATA    1150
Am4      ACAATTAACTCTACTGCTATTCTTTTTCTAACACTAGGTTTTAAAGTACCTGTGTCTATA    1261
14       ACAATTAACTCTACTGATATTCTTTTTCTAACACTAGGTTTTAATGTACCTGTGTCTATA    1035
27       ACAATTAACTCTACTGCTATTCTTTTTCTAACACTAGGTTTTAGAGTACCTGTGTCTATA    1216
C2       ACCATTAATTCTACTGCTATTCTTTTTCTAAAGCTAGGCTTTAAAGTACCTGTGTCTATA    1277
bIL67    ACAATTAACTCTACTGCTATACGTTTTCTAACACTAGGTTTTAAAGTACCTGTGTCTATA    1402
94p4     ACAATTAATTCTACTGCTATTCTTTTTCTAACGCTAGGTTTTAAAGTACCTGTGTCAATA    1283
12       ACAATTAATTCTACTGCTATTCTTTTTCTAACACTAGGCCTTAATGTACCAGTATCAATG    1155
E1       ACAATTAACTCTACTGATATTCTTTTTCTAACACTAGGTTTTAATGTACCTGTGTCTATA    1208

         <---D1     <-----D1
p6/4     AACTCTAGTGCCAAACTAAGTAACTGCATAAATACTACTAAACCAATCGCTTTCAATGTT    1154
A3       AACTCTAGTGCCAAACTAAGTAACTGCATAAATACAACTAAACCAATCGCTTTTAATGTT    1266
L81      AACTCTAGTATTAAACTAAGAACCTGCATAAAAACAACTAACCCAATTTCTTTCAATGTT    1277
D4       AACTCTAGTATTAAACTAAGAACCTGCATAAAAACAACTAACCCAATTTCTTTCAATGTT    1210
Am4      AACTCTAGTGCCAAACTAAGTAACTGCATAAATACAACTAAGCCAATTGCTTTCAATGTT    1321
14       AACTCTAGTGCTAAACTAAGTAACTGCATAAATACAACTAAGCCAATCGCTTTCAATGTT    1095
27       AACTCTAGTGCTAAACTAAGTAACTGCATAAATACAACTAAGCCAATCGCTTTCAATGTT    1276
C2       AACTCTAGTGCTAAACTAAGCAACTGCATAAATACAACTAAGCCAATCGCTCTCAATGTT    1337
bIL67    AACTCTAGTGCTAAACTAAGTAACTGCATAAATACAACTAAGCCAATCGCTCTCAATGTT    1462
94p4     AACTCTAGTATTAAACTAAGAACCTGCATAAAAATAACCAACCCAATTTCTTTCAATGTT    1343
12       AACTCTAGTACTAAACTAAGTAACTGCATAAATACAACTAAGCCAATTGCTTTCAATGTT    1169
E1       AACTCTAGTGCTAAACTAAGTAACTGCATAAATACAACTAAGCCAATCGCTTTCAATGTT    1268

                          R1
p6/4     TCAATCATTATCTTTCTATCTCCTTTATTATTAGTTTTCTATCTCGTATGTTTGTAGTGT    1214
A3       TCAATCATTATCTCTC-TTCTCCTTTATTAGTTTTCTATCTCGTATGTTTGTAGTGTACC    1325
L81      TCAATCATTATCTTTT-TTCTCCTTTATTATTAGTTTTCTATCTCATAAGTTTGTAGTGT    1336
D4       TCAATCATTATCTTTT-TTCTCCTTTATTATTAGTTTTCTATCTCATAAGTTTGTAGTGT    1269
Am4      TCAATCATTATCTTTC-TTCTCCTTTATTAGTTTTCTAATTTGTACGTTTGTAGTTCTCC    1380
14       TCAATCATTATCTTTCTTTCTCCTTTATTATTAGTTTTCTAATTGGTATGTTTGTAGTTC    1155
27       TCAATCATTATCTTTCTTTCTCCTTTATTATTAGTTTTCTAATTGGTATGTTTGTAGTTC    1336
C2       TCAATCATTATCTTTCTTTCTTCTTTCTTATTAG--------------------------    1371
bIL67    TCAATCATCATCTCTC-TTCTCCTTTATTATTAGTT-------------------------    1497
94p4     TCAATCACTACCTTTC-TTCTCCTCTATTAGTTTTCTATTTCGTAAGTTTGTAATTCTCC    1402
12       TCAATCATTATCTTTCTTTCTCCTTTATTATTAGTTTTCTATCTCATAAGTTTGTAGTGT    1229
E1       TCAATCATTATCTTTCTTTCTCCTTTATTATTAGTTTTCTATCTCATAAGTTTGTAGTGT    1328
```

Supplementary Figure S2. Average nucleotide identity (ANI) comparison between *Ceduovirus* phages. The average nucleotide identity (ANI) of the phage genomes was calculated with the ANI calculator [2] and OrthoANIu algorithm which uses USEARCH [3]. White boxes mark ANI values >80% to 85%, increasing gradient gray boxes from light to dark indicate ANI values in the following ranges: >85% to 90%, >90% to 95% and >95%.

		**Bacteriophages**									
	**Accession**	**12**	**14**	**27**	**94p4**	**P6/4**	**E1**	**L81**	**A3**	**D4**	**Am4**
M6202	KX373690	87.40	93.84	93.74	85.28	87.33	93.16	86.59	86.03	86.74	90.56
M6162	NC_031002	85.68	92.20	91.41	85.12	87.74	91.20	85.33	84.79	85.47	89.36
M6653	KX373691	87.27	92.91	93.09	84.34	87.33	93.49	86.64	86.08	86.75	90.27
M6654	KX373692	86.55	92.04	92.14	86.18	86.83	91.29	86.79	87.28	86.89	90.46
M6165	NC_031044	87.54	92.30	91.64	84.00	87.78	91.99	85.63	85.44	85.80	89.67
D4410	NC_031071	84.13	89.96	89.92	86.45	85.94	86.25	87.46	87.01	87.38	90.17
**14**	MH779519	82.25	X	97.58	85.60	90.34	91.67	85.92	87.19	87.49	91.63
**27**	MH779520	83.38	97.58	X	85.28	90.12	92.64	86.28	87.65	87.91	91.59
bIL67	NC_001629	87.40	93.19	93.19	85.52	88.06	93.01	86.60	86.34	86.73	91.23
**Am4**	MH779523	87.84	91.63	91.59	85.69	87.65	91.11	88.14	88.41	88.31	X
CHPC1182	MN689510	85.61	91.40	91.07	85.48	87.76	89.35	87.56	87.71	87.84	92.93
62402	MF443119	83.94	90.47	90.50	84.57	89.94	88.13	89.52	89.06	89.85	91.59
62403	MF443120	82.30	90.98	91.13	85.48	89.89	87.42	87.29	87.02	87.45	92.49
CHPC1020	MN689505	86.27	91.25	91.39	84.34	85.42	91.51	84.50	83.76	84.56	89.03
**94p4**	MH779521	88.82	85.60	85.28	X	85.26	85.65	91.05	90.99	91.12	85.69
M5938	NC_031075	88.28	92.99	92.03	85.41	87.59	93.43	87.81	87.26	90.73	89.84
D4412	NC_031009	87.80	86.45	86.67	87.21	85.94	90.30	87.42	87.01	87.34	89.91
**E1**	MH779525	87.44	91.67	92.64	85.65	91.06	X	88.39	89.22	89.67	91.11
5802	MF443126	89.40	89.28	89.51	85.62	87.98	91.09	87.33	87.51	87.56	87.99
**p6/4**	MH779527	88.07	90.34	90.12	85.26	X	91.06	85.79	85.68	85.94	87.65
CHPC967	MN689527	87.64	88.29	88.60	86.33	87.91	90.00	88.68	87.85	88.67	89.68
PC_S1	MN689533	91.51	85.70	85.78	90.89	86.51	88.64	91.34	91.90	91.68	83.89
CHPC134	MN689515	92.37	86.57	86.49	91.20	86.44	88.94	91.44	92.03	91.79	86.99
PC_B3	MN689532	92.20	87.13	87.26	91.15	86.40	88.92	91.50	92.09	91.85	86.88
5171F	MN689503	92.20	82.34	83.01	90.96	84.16	86.33	92.06	91.77	92.00	86.54
5205F	MN689504	89.50	80.29	80.51	90.34	83.62	84.57	91.02	91.07	91.17	85.05
PC_B1	MN689531	91.87	87.47	87.42	90.94	84.96	90.50	91.81	92.36	92.05	89.25
62606	MF443123	92.97	86.77	86.65	90.49	87.29	88.15	92.78	93.03	93.18	85.99
c2	NC_001706	90.59	84.76	84.73	90.42	87.63	87.21	90.90	90.61	91.16	87.64
**D4**	MH779524	92.68	87.49	87.91	91.12	85.94	89.67	99.36	97.80	X	88.31
**L81**	MH779526	92.69	85.92	86.28	91.05	85.79	88.39	X	97.33	99.36	88.14
**A3**	MH779522	92.19	87.19	87.65	90.99	85.68	89.22	97.33	X	97.80	88.41
CHPC1161	MN689506	95.12	85.99	86.18	89.70	85.98	89.45	92.08	91.91	91.54	85.81
vB_LacS_15	MN337887	93.56	86.32	86.51	89.03	87.05	90.09	91.28	91.01	90.73	89.11
**12**	MH779518	X	82.25	83.38	88.82	90.89	87.44	92.69	92.19	92.68	87.84
CHPC1183	MN689511	93.23	80.83	80.71	89.53	85.33	84.39	89.27	88.64	89.69	86.39
50102	MF443125	91.01	86.11	86.00	89.93	87.95	89.48	90.33	90.47	90.51	89.73
CHPC973	MN689529	90.59	85.81	85.84	89.28	88.55	89.15	90.51	90.69	90.61	89.10
20R03M	MK301442	88.12	87.40	87.46	85.15	87.76	89.53	87.62	87.77	87.79	87.01
CW09	MK301443	88.13	87.40	87.46	85.14	87.76	89.53	87.61	87.75	87.78	87.01
50504	MF443124	88.45	86.44	86.65	86.58	86.54	88.57	89.51	88.63	89.39	85.56
CHPC122	MN689512	88.31	84.93	85.30	88.36	87.76	87.44	89.56	90.29	89.61	90.78
CHPC972	MN689528	92.37	85.64	85.99	88.34	86.46	89.16	89.59	88.81	89.53	89.73
CHPC116	MN689507	86.86	86.38	86.53	84.68	88.39	89.17	87.57	87.45	87.79	88.13
CHPC1170	MN689508	89.37	90.67	90.35	85.67	88.88	91.03	88.40	88.16	88.48	89.16
37203	MF443121	87.85	86.43	86.50	87.21	85.81	86.08	86.52	85.99	86.75	88.41
74001	MF443122	89.30	87.27	87.39	86.02	85.16	88.58	87.13	86.51	87.20	87.49

Supplementary Figure S3. Maximum likelihood trees of *Ceduovirus* phages based on individual phage proteins; GP2 (A), GP4 (B), GP14 (C), DNApol (D), holin (E), lysin (F), ERF-like recombinase (G). Tree scales are given for the corresponding trees.

A

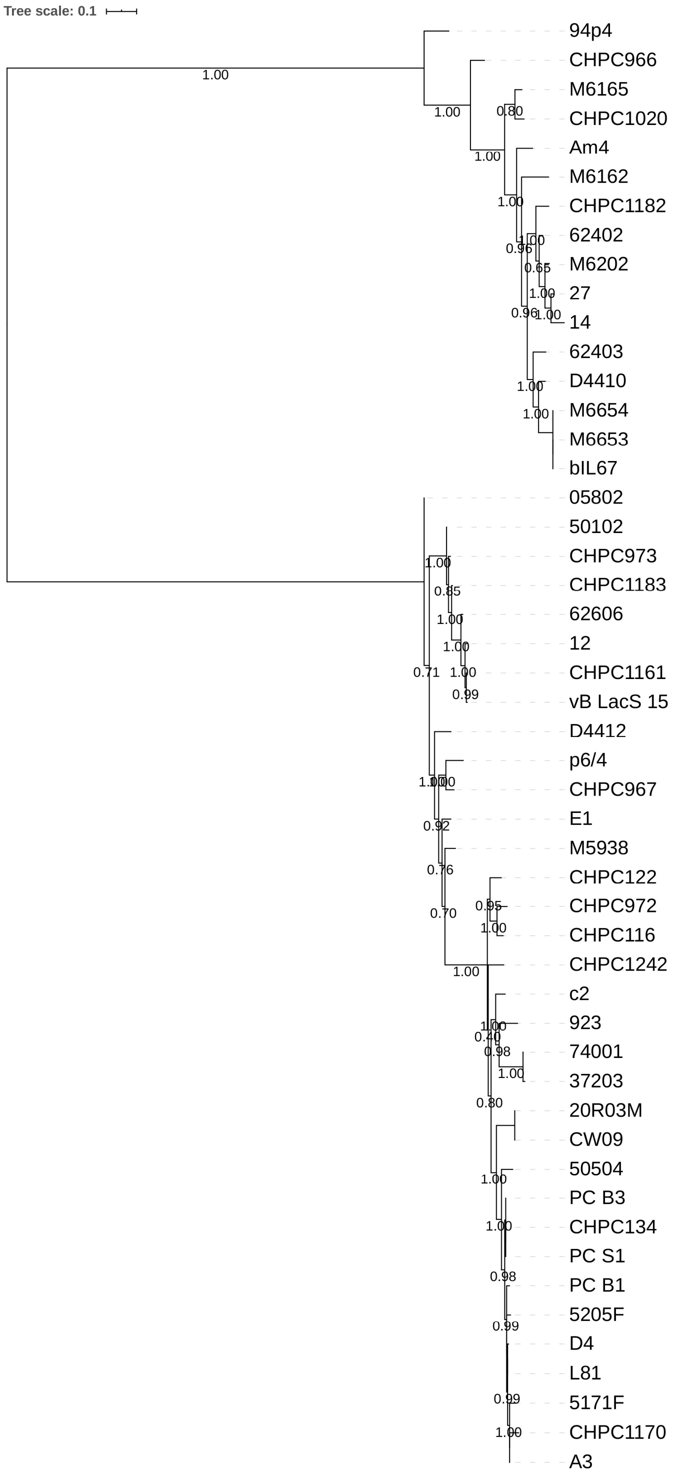

B

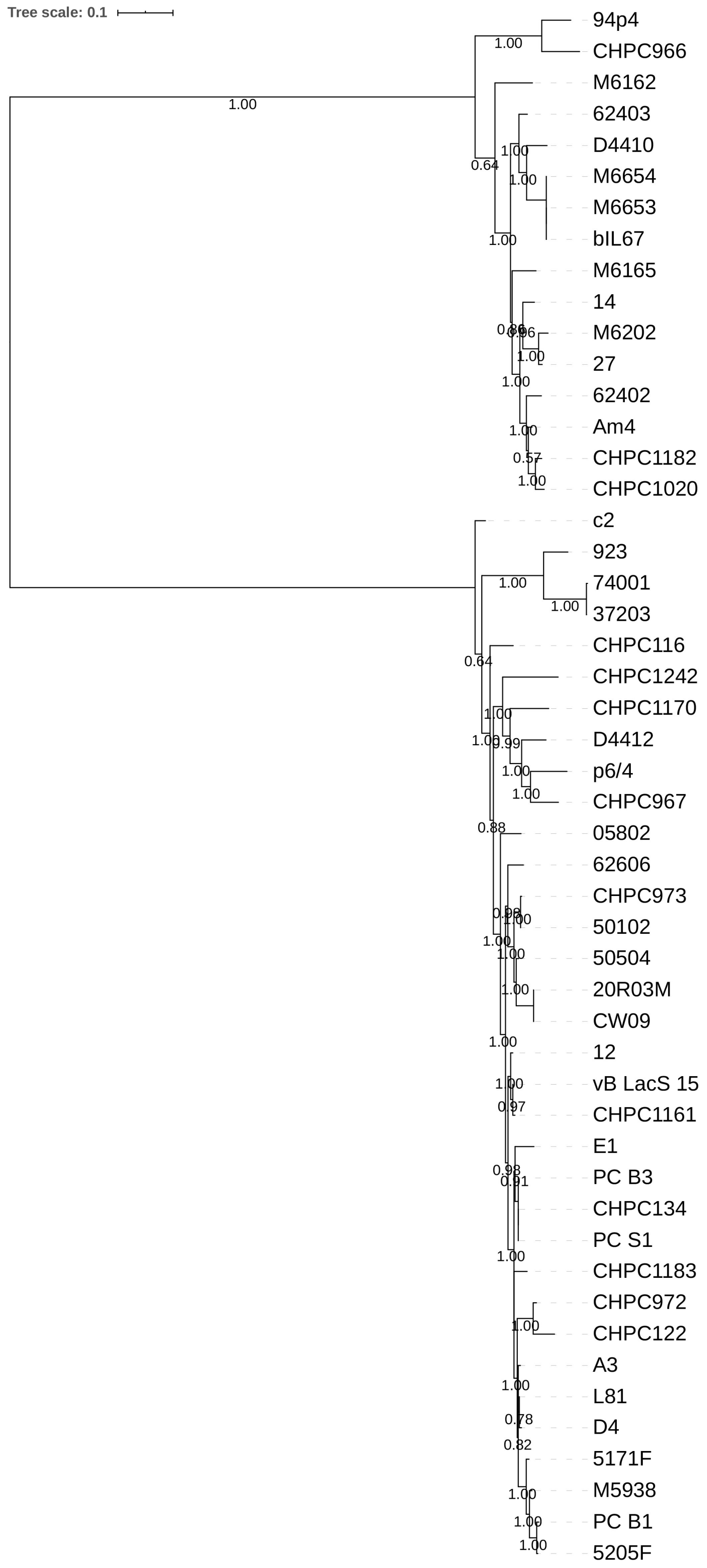

Tree scale: 0.1
94p4
CHPC966
M6162
62403
D4410
M6654
M6653
bIL67
M6165
14
M6202
27
62402
Am4
CHPC1182
CHPC1020
c2
923
74001
37203
CHPC116
CHPC1242
CHPC1170
D4412
p6/4
CHPC967
05802
62606
CHPC973
50102
50504
20R03M
CW09
12
vB LacS 15
CHPC1161
E1
PC B3
CHPC134
PC S1
CHPC1183
CHPC972
CHPC122
A3
L81
D4
5171F
M5938
PC B1
5205F

C

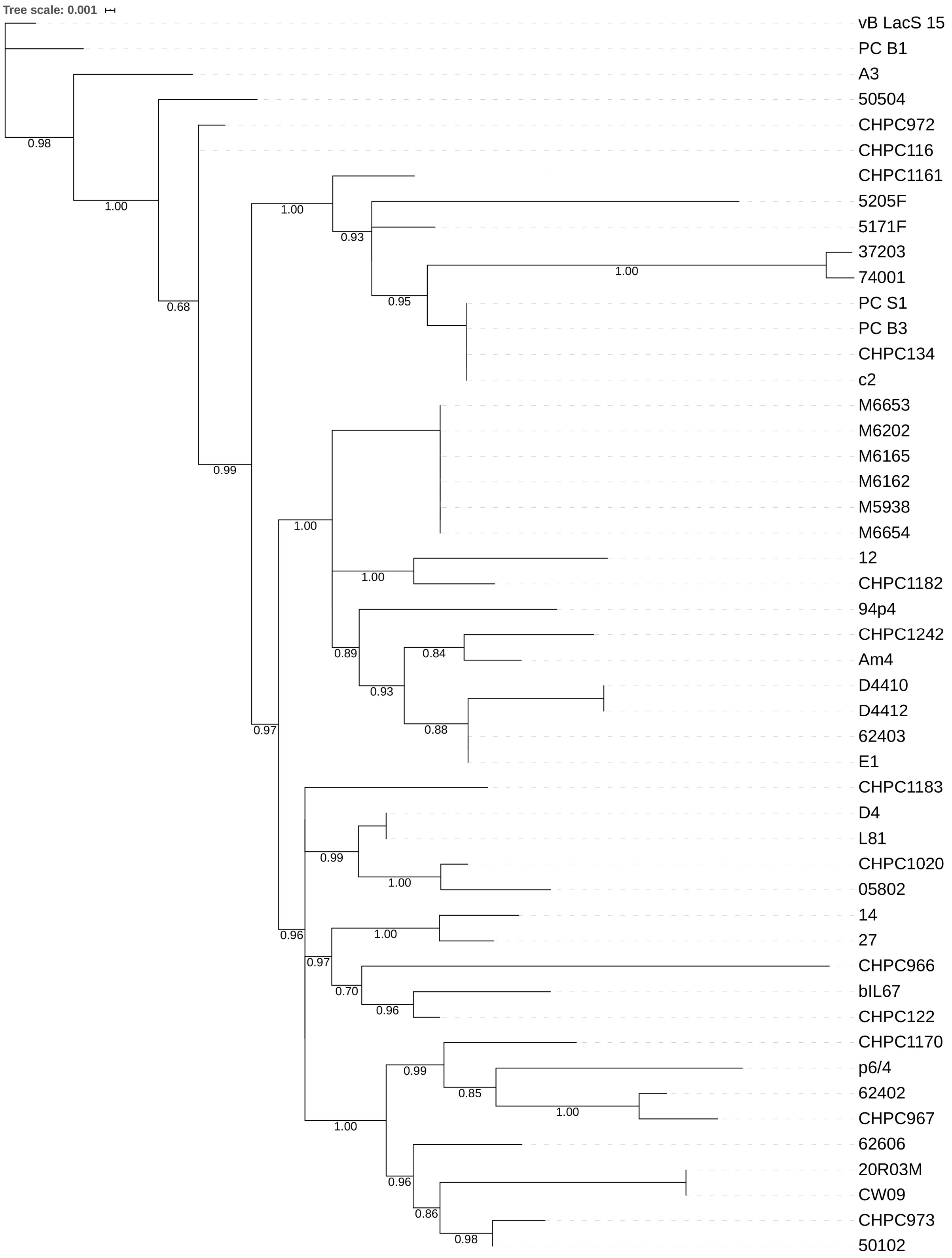
Tree scale: 0.001
vB LacS 15
PC B1
A3
50504
CHPC972
CHPC116
CHPC1161
5205F
5171F
37203
74001
PC S1
PC B3
CHPC134
c2
M6653
M6202
M6165
M6162
M5938
M6654
12
CHPC1182
94p4
CHPC1242
Am4
D4410
D4412
62403
E1
CHPC1183
D4
L81
CHPC1020
05802
14
27
CHPC966
bIL67
CHPC122
CHPC1170
p6/4
62402
CHPC967
62606
20R03M
CW09
CHPC973
50102
0.98
1.00
0.68
0.99
1.00
0.93
1.00
0.95
1.00
1.00
0.89
0.84
0.93
0.88
0.97
0.99
1.00
0.96
1.00
0.97
0.70
0.96
0.99
0.85
1.00
1.00
0.96
0.86
0.98

D

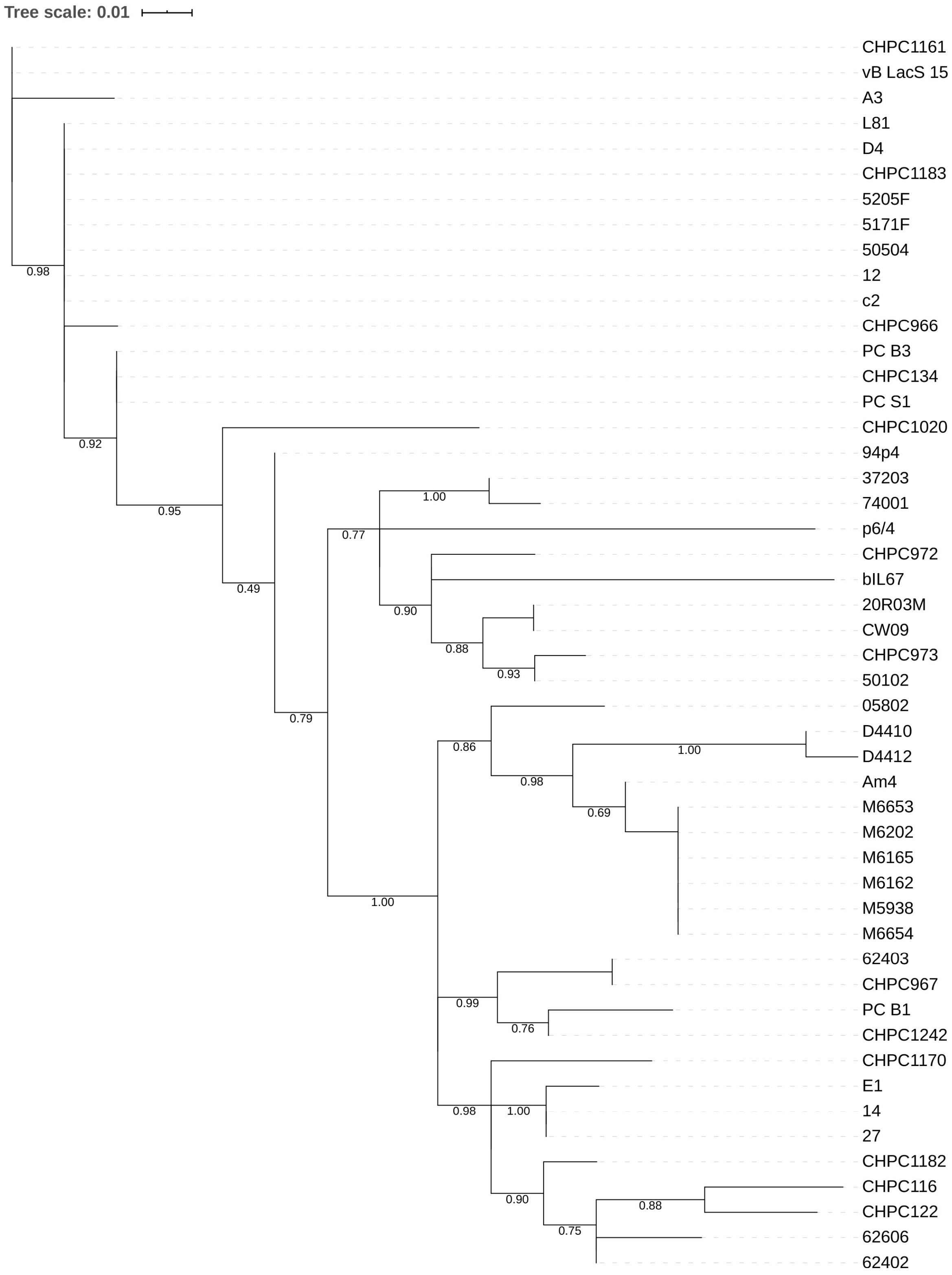
Tree scale: 0.01
CHPC1161
vB LacS 15
A3
L81
D4
CHPC1183
5205F
5171F
50504
12
c2
CHPC966
PC B3
CHPC134
PC S1
CHPC1020
94p4
37203
74001
p6/4
CHPC972
bIL67
20R03M
CW09
CHPC973
50102
05802
D4410
D4412
Am4
M6653
M6202
M6165
M6162
M5938
M6654
62403
CHPC967
PC B1
CHPC1242
CHPC1170
E1
14
27
CHPC1182
CHPC116
CHPC122
62606
62402
0.98
0.92
0.95
0.49
0.79
0.77
1.00
0.90
0.88
0.93
0.86
0.98
1.00
0.69
1.00
0.99
0.76
0.98
1.00
0.90
0.88
0.75

E

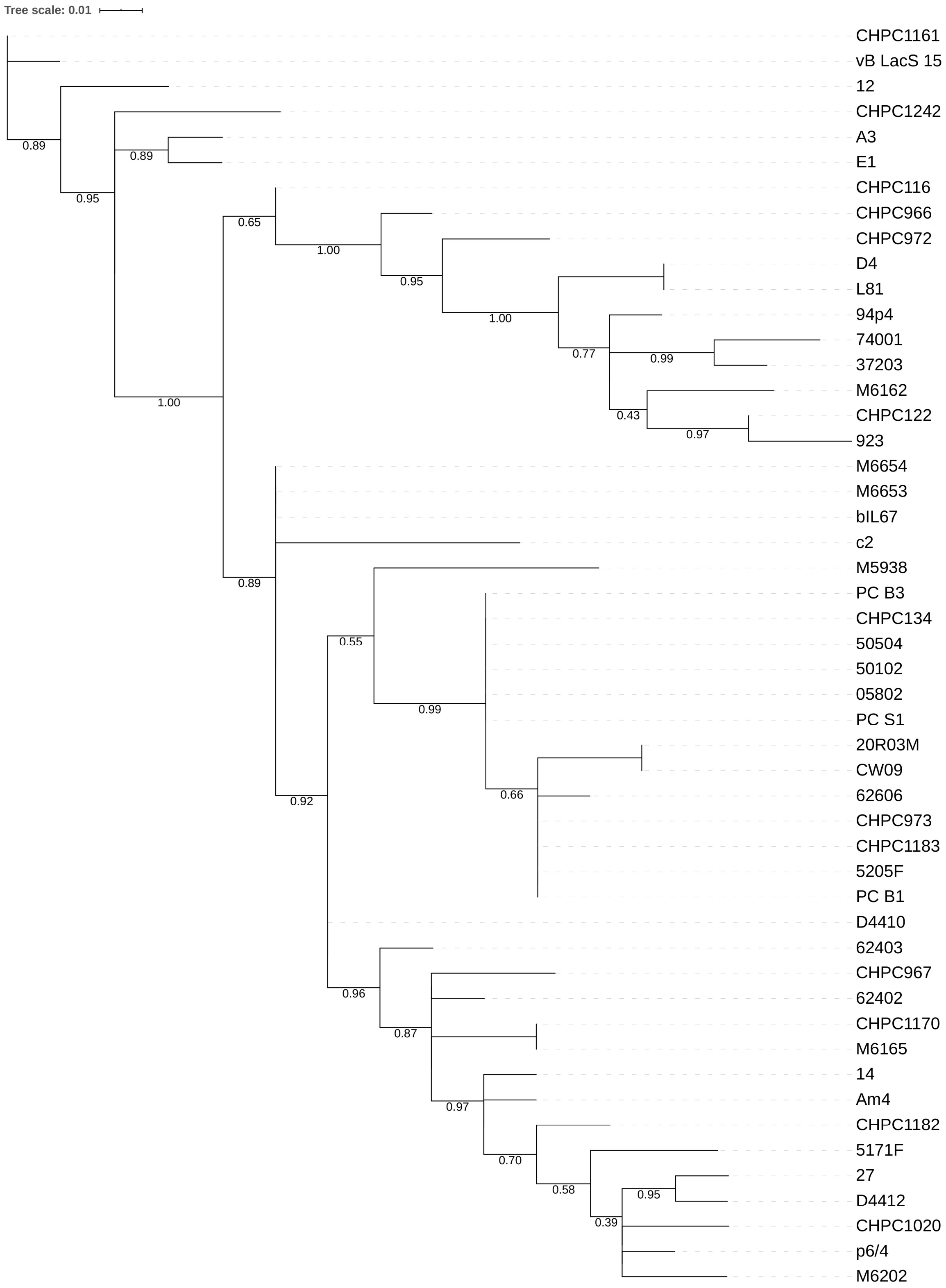
Tree scale: 0.01
CHPC1161
vB LacS 15
12
CHPC1242
A3
E1
CHPC116
CHPC966
CHPC972
D4
L81
94p4
74001
37203
M6162
CHPC122
923
M6654
M6653
bIL67
c2
M5938
PC B3
CHPC134
50504
50102
05802
PC S1
20R03M
CW09
62606
CHPC973
CHPC1183
5205F
PC B1
D4410
62403
CHPC967
62402
CHPC1170
M6165
14
Am4
CHPC1182
5171F
27
D4412
CHPC1020
p6/4
M6202

F

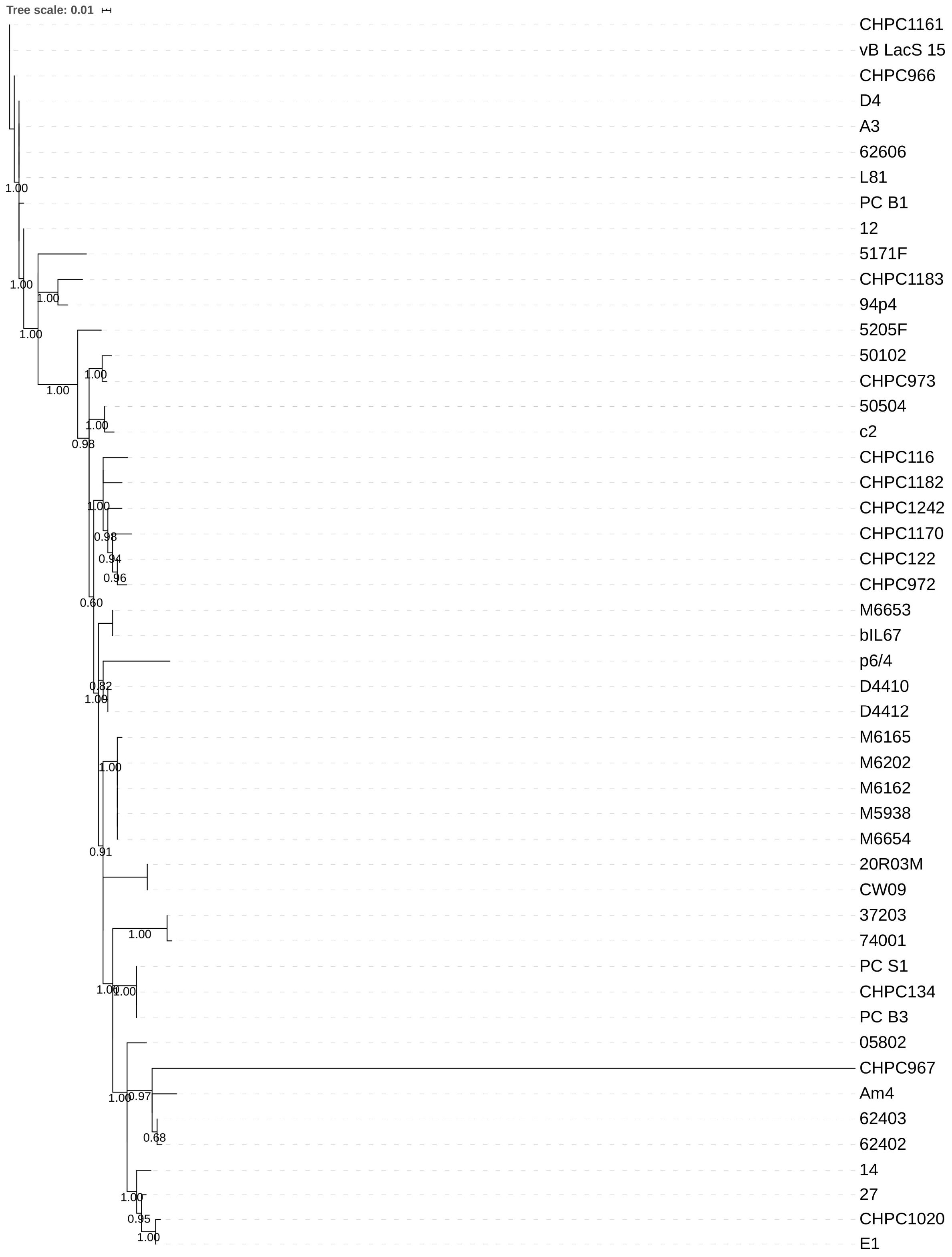

Tree scale: 0.01
CHPC1161
vB LacS 15
CHPC966
D4
A3
62606
L81
PC B1
12
5171F
CHPC1183
94p4
5205F
50102
CHPC973
50504
c2
CHPC116
CHPC1182
CHPC1242
CHPC1170
CHPC122
CHPC972
M6653
bIL67
p6/4
D4410
D4412
M6165
M6202
M6162
M5938
M6654
20R03M
CW09
37203
74001
PC S1
CHPC134
PC B3
05802
CHPC967
Am4
62403
62402
14
27
CHPC1020
E1
1.00
1.00
1.00
1.00
1.00
1.00
1.00
0.98
1.00
0.98
0.94
0.96
0.60
0.82
1.00
1.00
0.91
1.00
1.00
1.00
1.00
0.97
0.68
1.00
0.95
1.00

G

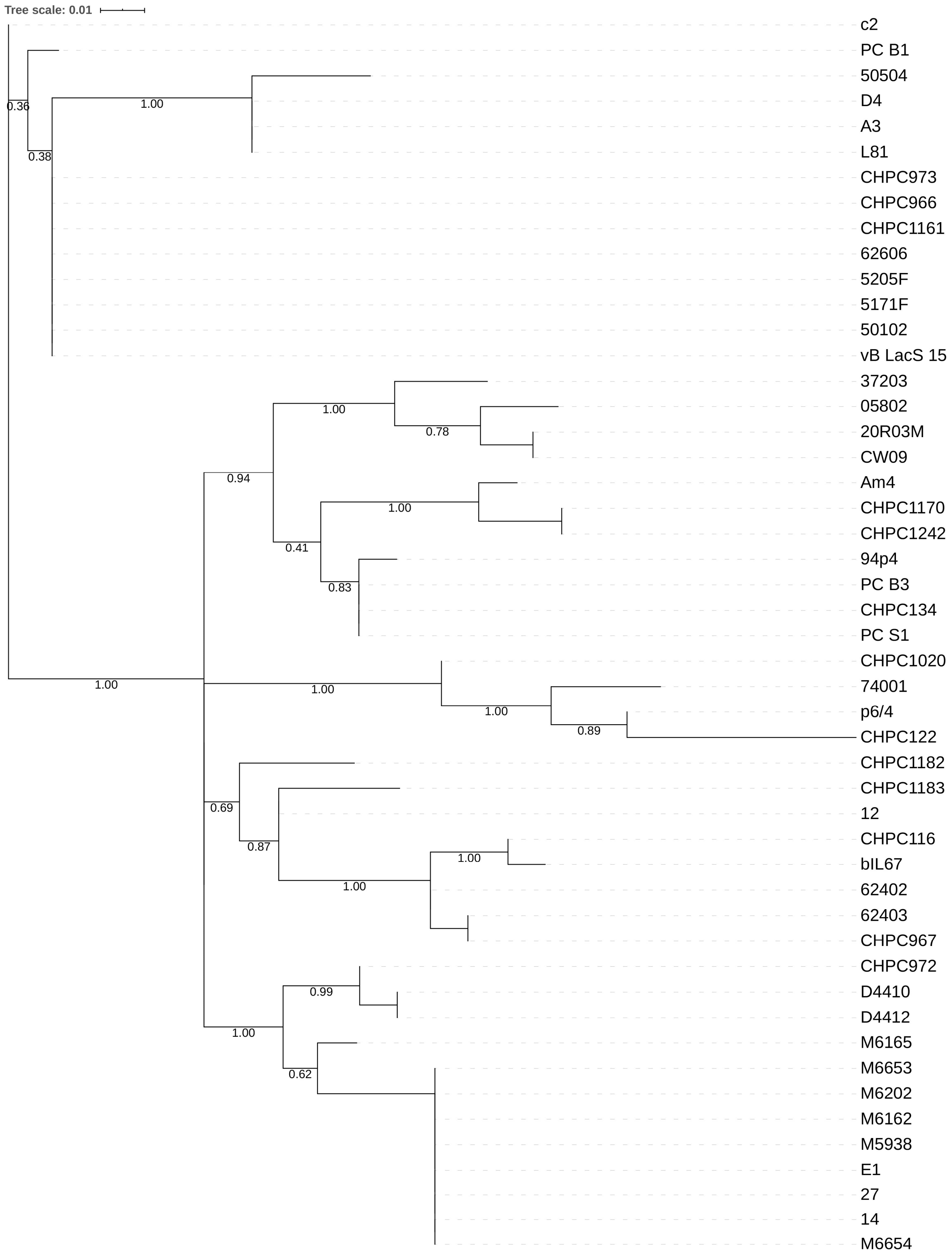
Tree scale: 0.01
c2
PC B1
50504
D4
A3
L81
CHPC973
CHPC966
CHPC1161
62606
5205F
5171F
50102
vB LacS 15
37203
05802
20R03M
CW09
Am4
CHPC1170
CHPC1242
94p4
PC B3
CHPC134
PC S1
CHPC1020
74001
p6/4
CHPC122
CHPC1182
CHPC1183
12
CHPC116
bIL67
62402
62403
CHPC967
CHPC972
D4410
D4412
M6165
M6653
M6202
M6162
M5938
E1
27
14
M6654
0.36
1.00
0.38
1.00
0.78
0.94
1.00
0.41
0.83
1.00
1.00
1.00
0.89
0.69
0.87
1.00
1.00
0.99
1.00
0.62

Supplementary Figure S4. Multiple alignment of GP3 protein sequences of phages sequenced in this study to analogous gene products of other *Ceduovirus* phages (protein id given in brackets). Similar residues are coloured based on their conservation according to BLOSUM62 scoring (max: 3.0, light blue; low: 0.5, gray).

```
D4412 (YP_009278611.1)-MiSWLNFEELLIHNPIELINfSKsnIqVAlSKKQYIdFFSNKavYmGLYYDEEMDFC
p6/4 (AXY83882)     ---MiSWLNFEELLIHNPIELINfSKsnIqVAlSKKQYIdFFSNKavYmGLYYDEEMDFC
12 (AXY83548)       ---MiSWLNFEELLIHNPIELINfSnssIqVAlSKKQYIdFFSNKavYmGLYYDEEMDFC
D4 (AXY83771)       ---MiSWLNFEELLIHNPIELINfSKssIqVAlSKKQYIdFFSNKavYmGLYYDEEMDFC
L81 (AXY83843)      ---MiSWLNFEELLIHNPIELINfSKssIqVAlSKKQYIdFFSNKavYmGLYYDEEMDFC
A3 (AXY83695)       ---MiSWLNFEELLIHNPIELINfSKssIqVAlSKKQYIdFFSNKavYmGLYYDEEMDFC
c2 (NP_043558.1)    ---MiSWLNFEELLIHNPIELINPSKDTIrVAMSqKQYIEFFSNKYTYNGLYYDEEMDFC
E1 (AXY83806)       ---MFSWLNFEELLIHNPIELINPSKDTISVAMSKKQYIEFFSNKYTYNGvYYDEEMDFC
M6202 (ANM46894.1)  ---MFSWLNFEELLIHNPIELINPSKDTmSVtMnKKQYIEFFSNKYTYNGvYYDEEMDFC
Am4 (AXY83733)   ---MNKMFSWLNFEELLIHNPIELINPSKDTISVAMSKKQYIEFFSNKYTYNGLYYDEEMDFC
M5938(YP_009287674.1)--MFSWLNFEELLIHNPIELINPSKDTISVAMnKKQYIEFFSNKYTYNGLYYDEEMDFC
M6653 (ANM46932.1)--MNKMFSWLNFEELLIHNPIELINPSKDTISVAMnKKQYIEFFSNKYTYNGLYYDEEMDFC
M6654 (ANM46971.1)--MNKMFSWLNFEELLIHNPIELINPSKDTISVAMnKKQYIEFFSNKYTYNGLYYDEEMDFC
bIL67 (NP_042342.1)--MNKMFSWLNFEELLIHNPIELINPSKDTISVAMnKKQYIEFFSNKYTYNGLYYDEEMDFC
M6162 (YP_009277872.1)--MFSWLNFEELLIHNPIELINPSKDTISVAMSKKQYIEFFSNKYTYNGLYYDEEMDFC
M6165 (YP_009283771.1)--MFSWLNFEELLIHNPIELINPSKDTISVAMnKKQYIEFFSNKYTYNGLYYDEEMDFC
D4410 (YP_009287215.1)--MFSWLNFEELLIHNPIdLINPSKDTISVAMnKKQYIEFFSNKYTYNGLYYDEEMDFC
14 (AXY83584)       ---MFSWLNFEELLIHNPIELINPSKDTISVAMSKKQYIEFFSNKYTYNGvYYDEEMDFC
27 (AXY83620)       ---MFSWLNFEELLIHNPIELINPSKDTISVAMSKKQYIEFFSNKYTYNGvYYDEEMDFC
94p4 (AXY83657)     ---MFSWLNFEELLIHNPIELINPSKDTISVAMSKKQYIEFFSNKYTYNGLYYDEEMDFC
CHL92 (AAO15642.1)  ---MFSWLNFEELLIHNPIELINPSKDTISVAMnKKQYIdFFSNKYTYNGvYYDEEMDFC

D4412        vmfYADPLQSsKnGemYAeGYIDVdMKIYRVKvLsNVSI---------------------
p6/4         vmfYADPLQSsKsGemYAeGYIDVdMKIYRiKvLsNVSI---------------------
12           vmfYADPLQSsKsGemYveGYIDVdMKIYRVKvLsNVym---------------------
D4           vmfYADPLQSsKsGemYveGYIDVdMKIYRVKvLsNVym---------------------
L81          vmfYADPLQSsKsGemYveGYIDVdMKIYRVKvLsNVym---------------------
A3           vmfYADPLQSsKsGemYveGYIDVdMKIYRVKvLsNVym---------------------
c2           LFYYADPLQSyKeGDVYAQGYIDVEMKIYRVKWLCNVSI---------------------
E1           LFYYADPLQSyKeGDmYAQeYIDVEMKIYRVKWLCNVSI---------------------
M6202        LFYYADPLQSyKeGDVYAQGYIDVEMKIYRVKWLCNVSI---------------------
Am4          LFYYADPLQSyKeGDVYAQGYIDVEMKIYRVKWLCNVSIklplapettgnlldvkdgagf
M5938        LFYYADPLQSVKDGDVYAQGYIDVEMKIYRVKWLCNVSI---------------------
M6653        LFYYADPLQSVKDGDVYAQGYIDVEMKIYRVKWLCNVSI---------------------
M6654        LFYYADPLQSVKDGDVYAQGYIDVEMKIYRVKWLCNVSI---------------------
bIL67        LFYYADPLQSVKDGDVYAQGYIDVEMKIYRVKWLCNVSI---------------------
M6162        LFYYADPLQSVKDGDIYAQGYIDVEMKIYRVKWLCNVSI---------------------
M6165        LFYYADPLQSVKDGDVYAQGYIDVEMKIYRVKWLCNVSI---------------------
D4410        LFYYADPLQSVKeGDVYAQGYIDVEMKIYRVKWLCNVSI---------------------
14           LFYYADPLQSyKeGDmYAQeYIDVEMKIYRVKWLCNVSI---------------------
27           LFYYADPLQSyKeGDmYAQeYIDVEMKIYRVKWLCNVSI---------------------
94p4         LFYYADPLQSaKDGDVYAQGYIDVEMKIYRVKWLCNVSI---------------------
CHL92        LFYYADPLQSyKDGDVYAQGYIDVEMKIYRVKWLCNVSI---------------------

D4412        ------------------------------------------------------------
p6/4         ------------------------------------------------------------
12           ------------------------------------------------------------
D4           ------------------------------------------------------------
L81          ------------------------------------------------------------
A3           ------------------------------------------------------------
c2           ------------------------------------------------------------
```

```
E1          ------------------------------------------------------------
M6202       ------------------------------------------------------------
Am4         sgtgngqsnmgigwfhfnewkkisqvfkagdkitisceiaffdtelysdsdaahvrlqia
M5938       ------------------------------------------------------------
M6653       ------------------------------------------------------------
M6654       ------------------------------------------------------------
bIL67       ------------------------------------------------------------
M6162       ------------------------------------------------------------
M6165       ------------------------------------------------------------
D4410       ------------------------------------------------------------
14          ------------------------------------------------------------
27          ------------------------------------------------------------
94p4        ------------------------------------------------------------
CHL92       ------------------------------------------------------------

D4412       ---------------------------SRFnSN---------------------------
p6/4        ---------------------------SRsnS----------------------------
12          ------------------------------------------------------------
D4          ------------------------------------------------------------
L81         ------------------------------------------------------------
A3          ------------------------------------------------------------
c2          ---------------------------SRpsgllq-------------------------
E1          ---------------------------nRF--NYN-------------------------
M6202       ---------------------------SRpdS----------------------------
Am4         ggnwrqlfrvdvknvngkfytreafktSeFteNpshivkvsqtmdidqdfiaqngdvnhi
M5938       ---------------------------SRFGSNsN-------------------------
M6653       ---------------------------SRF--NYN-------------------------
M6654       ---------------------------SRF--NYN-------------------------
bIL67       ---------------------------SRF--NYN-------------------------
M6162       ---------------------------SR-------------------------------
M6165       ---------------------------SRFGSNsN-------------------------
D4410       ---------------------------SRFGSNsN-------------------------
14          ---------------------------nRF--NYN-------------------------
27          ---------------------------nRF--NYN-------------------------
94p4        ---------------------------SRp--N---------------------------
CHL92       ---------------------------SRFnpN---------------------------

D4412       ------------------------------------------------FNLLEGsKDFSs
p6/4        ------------------------------------------------FdetQGklqpdG
12          ---------------------------------------------------LkGsemvk-
D4          ---------------------------------------------------LkGsemvk-
L81         ---------------------------------------------------LkGsemvk-
A3          ---------------------------------------------------LkGsemvk-
c2          ------------ttdgtqgVpqeGgKytHtAWSYSADGtDRFSTVYPnlNLLnGTKDFnG
E1          ------------------------------------------------FNLLEGTKDFSG
M6202       ------------------------------------------------FdgtQGklqpdG
Am4         ffifdripnganiivndlkIelnGaEkkHiAYAYSSDGaDRFSTIYPrFNLLEGTKDFSG
M5938       ------------------------------------------------FNLLvGTatFdG
M6653       ------------------------------------------------FNLLEGTKDFSG
M6654       ------------------------------------------------FNLLEGTKDFSG
bIL67       ------------------------------------------------FNLLEGTKDFSG
M6162       ------------------------------------------------------------
M6165       ------------------------------------------------FNLLvGTatFdG
D4410       ------------------------------------------------FNLLrGTakFdG
14          ------------------------------------------------FNLLEGTKDFSG
27          ------------------------------------------------FNLLEGTKDFSG
94p4        ------------------------------------------------------------
```

```
CHL92      ------------------------------------------------FNLLEGTKDFSG

D4412      fn-hNSsNns---vsTitkiklsgidntvmdVRtsgn--AfavGlYlYnAYsitaGqTvt
p6/4       kyKhtaWanikFLcndWdtfTlsKtSlvryGVdnRwTYKelQaGnYTaSfeLfGSdpaSg
12         ------------------------------------------------------------
D4         ------------------------------------------------------------
L81        ------------------------------------------------------------
A3         ------------------------------------------------------------
c2         dW-iNggvWgN--DGkYKGLTVKsyqkaWdGmfKKYi--vPQdGlYTWSsfVKsesDTSD
E1         aW-eySWNWtD--DGTFKGLaVKKtTgQWlGIRKaFi--APKdGVYTFSAYIKGSGDnAN
M6202      kyKhtaWanikFLcndWnefTlsKtSlvryGVdnRwTYKefQaGtYTaSfntfGSdpaSe
Am4        yWygeSWgWev--DGTFKGLTVKKrrhQWgGlyKvFT--APKnGVYTFSAYVKsSGDnAN
M5938      fk-pNSsN-ns--vsTitkiklsgidntvmdVRtsgn--AfavGlYlhnAYsitaGqTit
M6653      nW-drtggWtD--DGTYKGLvVKKkTtQWnGInKKFT--APKdGVYTFSAYIKaSGaTtS
M6654      nW-drtggWtD--DGTYKGLvVKKkTtQWnGInKKFT--APKdGVYTFSAYIKaSGaTtS
bIL67      nW-drtggWtD--DGTYKGLvVKKkTtQWnGInKKFT--APKdGVYTFSAYIKaSGaTtS
M6162      ------------------------------------------------------------
M6165      fk-pNSsN-ns--vsTitkiklsgidntvmdVRtsgn--AfavGlYlhnAYsitaGqTit
D4410      fn-pNSsN-ns--vsTitkiklsgidntvmdVRtsgn--AfavGlYlhnAYsitaGqTit
14         fW-gySWNWtD--DGTFKGLaVKKtTgQWlGIRKEFi--APKdGVYTFSAYIKGSGDnAN
27         aW-eySWNWtD--DGTFKGLaVKKtTgQWlGIRKaFi--APKdGVYTFSAYIKGSGDnAN
94p4       ------------------------------------------------------------
CHL92      dW-iNSssWlt--tGTYKnLTVKtqnvpWtaIsKKFT--vsipGtYTiSehVRniGsspv

D4412      IsfMArgtNdTKVLVGfeg--iangiKEFRLsPNwelytyTFTsTtsgthnFLv------
p6/4       -----------------------------------sWkKvelvsgfst------------
12         ------------------------------------------------------------
D4         ------------------------------------------------------------
L81        ------------------------------------------------------------
A3         ------------------------------------------------------------
c2         IfRVlfinNkefpiVG--------------LGhkFDWlRDsvTVpLKkGDevIf------
E1         IrRfvErwd----inGnwE--WVGDinDsfiGdNFDWkRDslTVnLKkGDklyp------
M6202      myKrAElvsefsfLcndwDtfmLsktslvRyGidghWvykvlqagtytanfYLfgsdpap
Am4        IiRytyknN---------D--YfGge---RiGnNFDWfRDsYTVTLKAGDtFIv------
M5938      IsfMArgtNGTKVLVGfeg--itnglKEFRLtPNwelythTFTaTtsgthnFLm------
M6653      ItRySning-----VGktE--WLkDs----LGnNFDWlRDTvTISLKAGqtaLf------
M6654      ItRySning-----VGktE--WLkDs----LGnNFDWlRDTvTISLKAGqtaLf------
bIL67      ItRySning-----VGktE--WLkDs----LGnNFDWlRDTvTISLKAGqtaLf------
M6162      --------------------------------PN--------------------------
M6165      IsfMArgtNGTKVLVGfeg--itnglKEFRLtPNwelythTFTaTtsgthnFLm------
D4410      IsfMArgtNGTKVLVGfeg--itnglKEFRLtPNwelythTFTaTtsgthnFLm------
14         IrRfvErwd----tnGnwE--WaGDinDsfiGdNFDWkRDslTVnLKkGDklyp------
27         IrRfvErwd----inGnrE--WVGDinDsfiGdNFDWkRDslTVnLKkGDklyt------
94p4       ------------------------------------------------------------
CHL92      qsyLi--------LnGkin--dakDd-----GtrFDWkvvsFTrTLsAGDvial------

D4412      --------------ygwdmva---GqwfQvynpKaEEGSiATPYMqaESEtTStDlPKW-
p6/4       --------------fYpttkvdrpshIgQytdttlEDsTnpnsYtweEaqedk----KW-
12         ---------------------------------------------------------eW-
D4         ---------------------------------------------------------KW-
L81        ---------------------------------------------------------KW-
A3         ---------------------------------------------------------KW-
c2         --------------nYgnlknNg-GkLs-vAGYKlEsGSiATPwMPSaSEvTtsDgPsyI
E1         --------------RYemAgt---GvLW-TAGHKWEEGdkATPYMPSESEATSVDFPKW-
M6202      gvwkkvelvsgfstfYpttksdrpshIgQytdtmlEDsTnpssYtwvEaqee-----KW-
Am4        --------------RYeitgwgvDtiLW-TAGHKWEEGSfATPYMPSESEtTksDrPsyI
M5938      --------------ygwdmda---GqwfQvynpKaEEGptATPYMqSESEtlSaDlPKW-
```

```
M6653           ---------------RYeiAsnNaDaiLW-TcGHKWEhGSvATqYMPSESEATSVDFPKW-
M6654           ---------------RYeiAsnNaDaiLW-TcGHKWEhGSvATqYMPSESEATSVDFPKW-
bIL67           ---------------RYeiAsnNaDaiLW-TcGHKWEhGSvATqYMPSESEATSVDFPKW-
M6162           ----------------------------------------------------------KW-
M6165           ---------------ygwdmda---GqwfQvynpKaEEGptATPYMqSESEtlSaDlPKW-
D4410           ---------------ygwdmda---GqwfQvynpKaElGynATsYMqSESESrh----KW-
14              ---------------RYeitgt---GvLW-TAGHKWEEGSvATPYMPSESEATSaDlPKW-
27              ---------------RYeiAgt---GvLW-TAGHKWEDGdkATPYMPSESEATSVDFPKW-
94p4            ----------------------------------------------------------KW-
CHL92           ---------------ethngtt---GqIs-vAGYKvEQGStATPYMPSESEtTSaDlPKW-

D4412           ---------------------------NVTKTgMrVsPqAKQITmVqaGtLmKCeiDnNI
p6/4            ---------------------------NVTKTgMLVsPqAKQITmVqsGALmrCeinnNI
12              ---------------------------aiTKTgMLVsPqtKQITmVqaGALmrCeinnNI
D4              ---------------------------aiTKTgMrVsPqAKQITmVqaGvLmrCeinnNI
L81             ---------------------------aiTKTgMrVsPqAKQITmVqaGvLmrCeinnNI
A3              ---------------------------aiTKTgMrVsPqAKQITmVqaGALmrCeinnNI
c2              GQYTDYTLEDSTNPSSYTWREIREDKwNVTKigMLVsPqdKQITmVqaGALmKCginndI
E1              ---------------------------NVTKTgMLVsPqtKQITmVqaGALmKCeinnNI
M6202           ---------------------------NVsKTEMIVNsKAKtITTVLNGALSKCTKDKdI
Am4             GQYTDYTLEDSTNPSSYTWREIQEDKlNVsKTEMVVNaKnKtITTVLNGALAKCTKDKdI
M5938           ---------------------------NVTKTgMLVsPqAKQITmVqaGALtrCeinnNI
M6653           ---------------------------NVsKTEMVVNPKtKaITTVLNGALAKCTKDKNI
M6654           ---------------------------NVsKTEMVVNPKtKaITTVLNGALAKCTKDKNI
bIL67           ---------------------------NVsKTEMVVNPKtKaITTVLNGALAKCTKDKNI
M6162           ---------------------------NVTKTEMIVstKtKtITTVLNGALAKCTKDKNI
M6165           ---------------------------NVTKTEMVVNaKnKtITnVLNGALAKCTKDKNI
D4410           ---------------------------NVsKTEMVVNaKnKtITTVLNGALAKCTKDKdv
14              ---------------------------NVsKTEMVVNtKAKtITTVLNGALSKCTKDKdI
27              ---------------------------NVsKTEMVVNaKtKtITTVLNGALSKCTKDKNI
94p4            ---------------------------NVsKTEMVVNsKAKtITTVLNGALAKCTKnKdv
CHL92           ---------------------------NVsKTEMVVNtKAKtITTVLNGALSKCTKDKdI

D4412           sGWTdGTtqlNYS-----------GqDFIIdGYGmRGLhNG
p6/4            TGWTdGTtqleYS-----------GqeFIIdGYGmRGLhNG
12              sGWTdGTtqlNcS-----------GqDFvIdGYGmRGLhNG
D4              TGWTdGTtqleYS-----------GqDFIIdGYGmRGLhNG
L81             TGWTdGTtqleYS-----------GqDFIIdGYGmRGLhNG
A3              TGWTdGTtqleYS-----------GqDFIIdGYGmRGLhNG
c2              TGWTdGTtqlNYS-----------GqDFIIdGYGmRGLhNG
E1              TGWTdGTtqlNYS-----------GqDFIIdGYGmRGLhNG
M6202           TGWrNlpPNeNYnYRQPQYSLDIGaDDFIISGYGLRGLKNG
Am4             TGWrNsqPNaNYnYRQPQYtLDIGaDDFIISGYGLRGLKNG
M5938           TGWTdGTtqleYS-----------GqDFIIdGYGmRGLhNG
M6653           TGWqNplPNgNYkYRQPQYSLDIGaDDFIISGfGLRGLKNG
M6654           TGWqNplPNgNYkYRQPQYSLDIGaDDFIISGfGLRGLKNG
bIL67           TGWqNpllteiin---------Idnrsil------------
M6162           TGWlNqptNaNYnYRQPQYSLDIesgDFIISGfGLRGLKNG
M6165           TGWkNlqPNaNYnYRQPQYSLDIGtDDFIISGfGLRGLKNG
D4410           TGWrNsqPNasYnYRQPQYSLDIGaDDFIISGfGLRGLKNG
14              TGWlNsqPNaNYnYRQPQYSLDIGaDDFIISGYGLRGLKNG
27              TGWgNpkPNasYkYqQPQYSLDIGvDDFIISGYGLRGLKNG
94p4            TGWqNsqPNtNYkYRQPfYSLDIGvDDFIISGYGLRGLKNG
CHL92           TGWlNsqPNaNYnYRQPQYSLDIGtDDFIISGYGLRGLKNG
```

Supplementary Figure S5. Maximum-likelihood phylogenetic tree of the putative tape measure proteins (GP8) of *Lactococcus Ceduovirus* phages sequenced in this study (in bold) with ORF31 of phage bIL67 (protei id: NP_042314.1), ORF*l10* of phage c2 (protein id: NP_043558.1), ORF5 of phage CHL92 (protein id: AAO15644.1), CHPC116_000134 (protein id: QGT52480.1) and CHPC134_000507 (protein id: QGT52852.1) as reference. Phylogenies were reconstructed from multiple alignments of complete amino acid sequences generated using MUSCLE with eight iterations (default settings) implemented within the Geneious 8.1 software [4]. A maximum-likelihood tree was constructed using PHYML and the LG substitution model with 100 bootstrap resamplings [44]. Evolutionary distance can be measured by the scale bar. Bootstrap proportions (%) are marked accordingly.

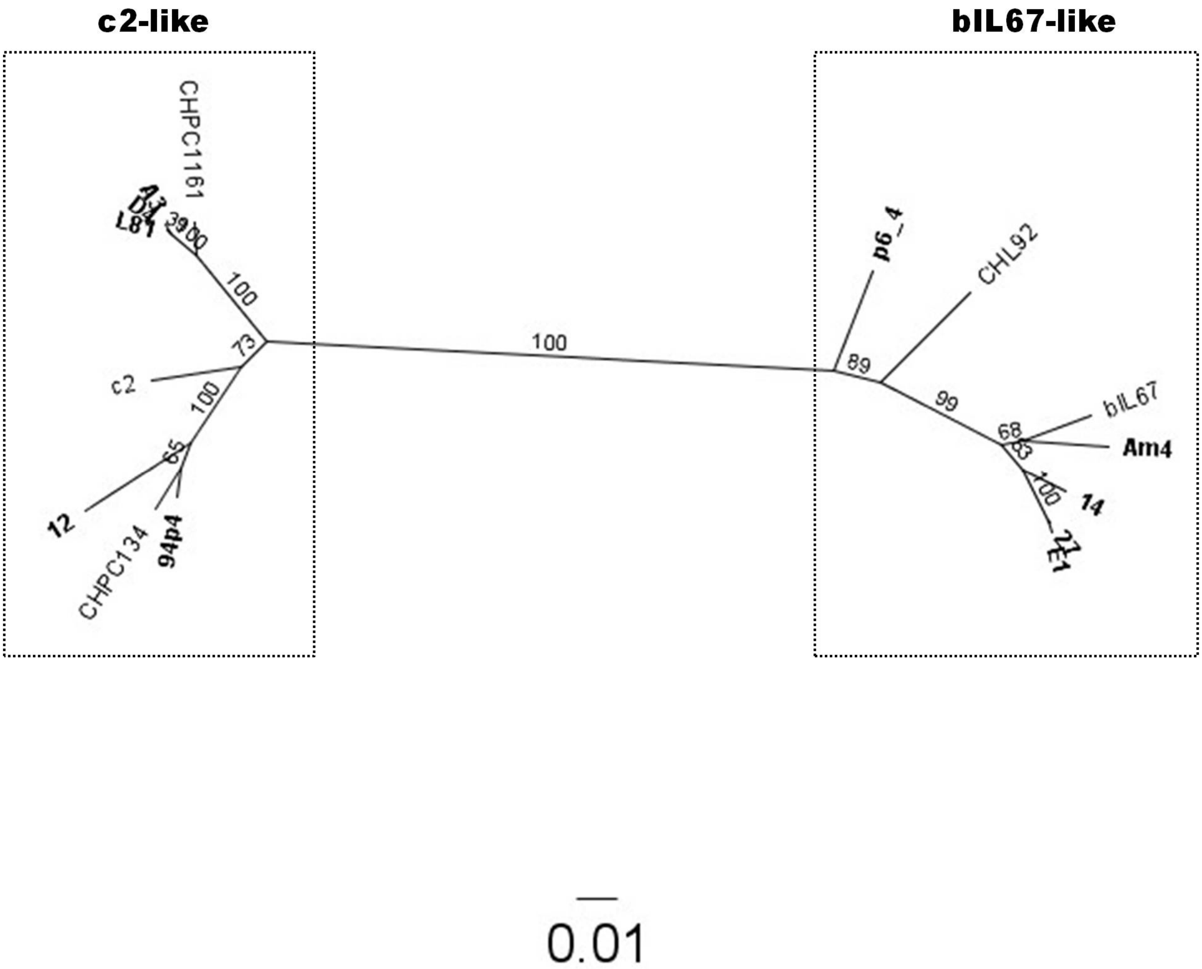

Supplementary Figure S6. Alignment of the non-coding intergenic region between late and early genes of studied *Ceduovirus* phages and reference phages c2, bIL67 and 923. Sequences were aligned using the ClustalOmega tool [1]. Underlined are the -35 and -10 boxes of the PE1 and PE2 promoters.

```
               5’ CCCGATATCCAATGTGTTTTTGTG 3’
               ←—— PE2   -35
p6/4        AAATCATGTTACAATTCAATGTGTTTTTGTGTTACTCTTTAATTACAAGA---------  81
923         AAGTCATGTTACAATTCAAGGTATTTTTATGTTACTCTTTAATTACAAGA---------  347

                5’CCGGAATTCCAAGCTATCAA-ATATTTC 3’
bIL67       AAGTTATATTACAATTCAAGCTATCAA-ATATTTCTTTTTAGTTACTTTATTTTAGACA  58
14          AAGTTATATTACAATTCAAGCTATCAA-ATATTTCTTTTTAATTACTTTATTTTAGGTA  89
E1          AAGTTATATTACAATTCAAGCTATCAA-ATATTTCTTTTTAGTTACCTTATTTTAGGTA  234
27          AAGTTATATTACAATTCAAGCTATCAA-ATATTTCTTTTTAATTACTTTATTTTAGGTA  123

                                   5’ CCGGAATTCTARTTACYTTGCTAAAGGG 3’
94p4        AAATAATATTACAATTCAAGCTATCAA-ATATTTCTTTTTAATTACTTTGCTAAAGGGT  112
Am4         AAATTATATTACAATTCAAGTTATCAA-ATATTTCTTTTTAGTTACCTTATTAAAGGGT  133
c6A         AAATTATATTACAATTCAAGCTATCAA-ATATTTCTTTTTAGTTACTTTGCTAAATGGT  358
D4          AAATAATATTACAATTCAAGCTATCAA-ATATTTCTTTTTAGTTACCTTGCTAAAGGGT  141
12          AAATAATATTACAATTCAAGCTATCAA-ATATTTCTTTTTAGTTACTTTGCTAAAGGGT  98
A3          AAATAATATTACAATTCAAGCTATCAA-ATATTTCTTTTTAGTTACCTTGCTAAAGGGT  91
L81         AAATAATATTACAATTCAAGCTATCAA-ATATTTCTTTTTAGTTACCTTGCTAAAGGGT  58
c2          AAATAATATTACAATTCAAGCTATCAA-ATATTTCTTTTTAGTTACCTTGCTAAAGGGT  58
            **.* **.***********  *.* :: .*.**:** ****.**** : .

p6/4        ATTTT---------AGACAGAGTTA-TCCCC-CCCCCGTCATGCAATTATTAGAGATTCT 130
923         ATTTT---------AGACACAGTTATTTCCC-CCCCCGTCATGCAATTAGTAGAGATTCT 397

bIL67       ATAATTGCCCTTGCCTTTTTGGTTAAGCCCCTAGCCCCTAACGTGTCTTGT--TATCCCA 116
14          ATAGTTGCCCTTGCCTTTTTGGTTAAGCCCCTAGCCCCTAACGTGTCTTGT--TATCCCA 147
E1          ATAGTTGCCCTTGCCTTTTTGGTTAAGCCCCTAGCCCCTAACGTGTCTTGT--TATCCCA 292
27          ATAGTTGCCCTTGCCTTTTTGGTTAAGCCCCTAGCCCCTAACGTGTCTTGT--TATCCCA 181

94p4        ATAACTATCCACGCAAACGCAGTTTTTTAT--CCCCCCTATTGAATCAATTAATATGTCA 170
Am4         ATAGTTATCCACGCAAACGCAGTTTTTAT-CCCCCCCCTCTTGAACTAATCAATCTGTCA 192
c6A         ATAACTACCCACGCAAACGCAGTTTTTA---TCCCCCCTCTTGAATTAATCAATATGTCA 415
D4          ATAACTACCCACGCAAACGCAGTTTTT--ATCCCCCCCTCTTGAACTAATTAATATGTCA 199
12          ATAACTACCCACGCAAACGCAAGTTTTTATCCCCCCCCTCTTGAATCAATTAATATGTCA 158
A3          ATAACTACCCACGCAAACGCAG-TTTTTATCCCCCCCCTCTTGAACTAATTAATATGTCA 150
L81         ATAACTACCCACGCAAACGCAG-TTTTTATCC-CCCCCTCTTGAACTAATTAATATGTCA 116
c2          ATAACTACCCACGCAAACGCAG-TTTTTATCC-CCCCCTCTTGAACTAATTAATATGTCA 116
            **:           . :   .. *:         *** *.: * .  ::    : :  *:

p6/4        G-------ATAAGGTAGTTTGCTTAG--------------------TTACTAGCAGTCAG 163
923         G-------ATAAGGTAGTTTGCTTAG--------------------TTATTAGCAGTCAG 430

bIL67       GCAACCTAAAGAACAAGTAAACAAATTTCATTCTTGTTATGTAATAGGTCTGTCAGACTT 176
14          GCAACCTAAAGAACAAGTAAACAAATTTCATTCTTGTTATATAATAGGTCTGTCAGACTT 207
E1          GCAACCTAAAGAACAAGTAAACAAATTTCATTCTTGTTATATAATAGGTCTGTCAGACTT 352
27          GCAACCTAAAGAACAAGTAAACAAATTTCATTCTTGTTATATAATAGGTCTGTCAGACTT 241

94p4        GC----------GCTAGTA---------------------------ACTCAATCAATCCT 193
Am4         GC----------GCTAGTA---------------------------ACTTAATCAGTCCT 215
c6A         GC----------GCTAGTA---------------------------ACTCAATCAATCCT 438
D4          GC----------GCTAGTA---------------------------ACTCAATCAGTCCT 222
```

```
12          GC----------GCTAGTA---------------------------ACTCAATTAATTCT 181
A3          GC----------GCTAGTA---------------------------ACTCAATCAGTCCT 173
L81         GC----------GCTAGTA---------------------------ACTCAATCAGTCCT 139
c2          GC----------GCTAGTA---------------------------ACTCAATCAGTCCT 139
            *           . :***:                             : :.  *.:

p6/4        CTCCGTTAATATAATTGCACCGCTCGTTTTGTTTTACGT------GAGTTCAAACTGGCA 217
923         CTCCGTTAATCTAATTGCACCGCTCGTTTTGTTTTACGT------GAGTTCAAACTGGCA 484

bIL67       CCAAG---------CGTCACGGAGTGTTTTAGTTCACGACACTCATGGAACTCACAAGAT 227
14          CCAAG---------CGTCACGGAGTGTTTTAGTTCACGACACTCATGGAACTCACAAGAT 258
E1          CCAAG---------CGTCACGGAGTGTTTTAGTTCACGACACTCATGGAACTCACAAGAT 403
27          CCAAG---------CGTCACGGAGTGTTTTAGTTCACGACACTCATGGAACTCACAAAAT 292

94p4        CACAT---------CAATTTGGCTA-----TG-------------ATGAACACCCAAGCG 226
Am4         CACAT---------CAATTCGGCTA-----TG-------------ATGAACACCCAAGCG 248
c6A         CACAT---------CAATTCGGCTA-----TG-------------ATGAACACCCAAGCG 471
D4          CACAT---------CAATTCGGCTA-----TG-------------ATGAACACCCAAGCG 255
12          CACAT---------CAATTAGGCTA-----TG-------------ATGAACACCCAAGCG 214
A3          CACAT---------CAATTCGGCTA-----TG-------------ATGAACACCCAAGCG 206
L81         CACAT---------CAATTCGGCTA-----TG-------------ATGAACACCCAAGCG 172
c2          CACAT---------CAATTCGGCTA-----TG-------------ATGAACACCCAAGCG 172
            * ..              :  *.                        *::*:..*:...

p6/4        TAAACAGAACACTCACAACCTGTCCAACTTTTTTATCAGT--TGGCAATTTCATATATAG 275
923         TAAACAGAATACTCACAACCTGTCCAACTTTTTTGTCAGT--TGGCAATTTCATATATAG 542

bIL67       TTCATTTAATGCTAACCTCTAG-CC---TTTTT-GTATGTTATTTCAATTTTCAATTAGT 282
14          TTCATTTAATGCTAACCTCTAG-CT---TTTTT-GTATGTTATTTCAATTTTCAATTAGT 313
E1          TTCATTTAATGCTAACCTCTAG-CC---TTTTT-GTATGTTATTTCAATTTTCAATTAGT 458
27          TTCATTTAATGCTAACCTCTAG-CT---TTTTT-GTATGTTATTTCAATTTTCAATTAGT 347

94p4        GTAACTTCTTATTTAAC-TTTG-CC---TATGTTGGGGGA----ACGTTT---------- 267
Am4         GTAACTTCTTATTTAAA-TTTG-CC---TATGTTGGGGGA----ACGTTT---------- 289
c6A         GTAACTTCTTATTTAAC-TTTG-CC---TATGTTGGGGGA----GCGTTT---------- 512
D4          GTAACTTCTTATTTAAC-TTTG-CC---TATGTTGGGGGA----GCGTTT---------- 296
12          GTAACTTCTTATTTAAC-TTTG-CC---TATGTTGGGGGA----GCGTTT---------- 255
A3          GTAACTTCTTATTTAAC-TTTG-CC---TATGTTGGGGGA----GCGTTT---------- 247
L81         GTAACTTCTTATTTAAC-TTTG-CC---TATGTTGGGGGA----GCGTTT---------- 213
c2          GTAACTTCTTATTTAAC-TTTG-CC---TATGTTGGGGGA----GCGTTT---------- 213
             :.* : .: . * *..   :* *    *:* * .   *:     *.:**

p6/4        CGACAAACTACGGCTACCAAATA--ACATAAAAGTTAATGCT-CCTTAA----CCGCTAT 328
923         CGACAAACTACGGCTACCAAATA--ACGTAATAGTTAATGCT-CCTTAA----CCGCTAT 595

bIL67       CGTCCTTT-TTAGCAACCATAGACAACTCA--AGGCAAATCTTGCAAAGACTTTCGATAA 339
14          CGTCCTTT-TTAGCAACCATAGACAACTCA--AGGCAAATCTTGCAAAGACTTTCGATAA 370
E1          CGTCCTTT-TTAGCAACCATAGACAACTCA--AGGCAAATCTTGCAAAGACTTTCGATAA 515
27          CGTCCTTT-TTAGCAACCATAGACAACTCA--AGGCAAATCTTGCAAAGACTTTCGATAA 404

94p4        ----GAAA-CTTGCTTTCAGTGACATCACACAGGGCTAC---CGCTTTGCCTAACTCATT 319
Am4         ----AGAA-CTTGCTTCCAGTGACATCACACAGGGCTAC---CGCTTTGCCTAACTCATT 341
c6A         ----GAAA-CTTGCTTTCAGTGACATCACACAGGGCTAC---CGCTTTGCCTAATTCATT 564
D4          ----GAAA-CTTGCTTTCAGTGACATCACACAGGGCTAC---CGCTTTGCCTAACTCATT 348
12          ----GAAA-CTTGCTTTCAGTGACATCACACAGGGCTAC---CGCTTTGCCTAACTCATT 307
A3          ----GAAA-CTTGCTTTCAGTGACATCACACAGGGCTAC---CGCTTTGCCTAACTCATT 299
L81         ----GAAA-CTTGCTTTCAGTGACATCACACAGGGCTAC---CGCTTTGCCTAACTCATT 265
```

```
c2         ----GAAA-CTTGCTTTCAGTGACATCACACAGGGCTAC---CGCTTTGCCTAACTCATT 265
                 :     **:: ** : *  :*  *  .*  :*      *:::.       .:::

p6/4       ATACTATCCTATTCATAAGGTCTTGTGGGGGTTTACAAGCTCAGGGAAATTTGCTTCATA 388
923        ATACTATCCTATTCTTAAGGGCTTGTGGG-GTTTACAAGCTCAGGGAAATATGCTTCATA 654

bIL67      TTACTAGCCT-A---TTCGGTCTAGT----Gttt-tctACTCCTAA---ACTGTCAACAA 387
14         TTACTAGCCT-A---TTCGGTCTAGT----Gttc-tctACTCCTAA---ACTGTCAACAA 418
E1         TTACTAGCCT-A---TTCGGTCTAGT----Gttc-tctACTCCTAA---ACTGTCAACAA 563
27         TTACTAGCCT-A---TTCGGTCTAGT----Gttc-tctACTCCTAA---ACTGTCAACAA 452

94p4       ACTCACGCCTTA---TTCAGTACGGT----TTTCATATACTCACTT---TC------TAA 363
Am4        ACTCACGCCTTA---TTCAGTACGGT----TTTCATATACTCACTT---TC------TAA 385
c6A        ACTTGCGCCTTA---TTCAGTACGGT----TTTCATATACTCAATT---TC------TAA 608
D4         ACTCGCGCCTTA---TTCAGTACGGT----TTTCATATACTCAATT---CC------TAA 392
12         ACTCACGCCTTA---TTCAGTACGGT----TTTCATATACTCGCTT---TC------TAA 351
A3         ACTCGCGCCTTA---TTCAGTACGGT----TTTCATATACTCAATT---CC------TAA 343
L81        ACTCGCGCCTTA---TTCAGTACGGT----TTTCATATACTCAATT---CC------TAA 309
c2         ACTCGCGCCTTA---TTCAGTACGGT----TTTCATATACTCAATT---CC------TAA 309
           : :  . *** :   *:..* .  **     **   .:.***                :*
                                             <---PE1 -10
p6/4       GGCTTCCGCCTCGCATTTT-AGATATATTCAATTTTCTATATATCTATTATAACATACGT 447
923        GGCTTTCGCCTCGCTTTTTTAGATATATTCAATTTTTTATATTTATATTATAACATACGT 714

bIL67      GTCATCAGCTA----AC---AGTCGTTA--AATTTTTGATATATTTATTATAGCATACGA 438
14         GTCATCAGCTA----AC---AGTCGTTA--AATTTTTATATATATTATTATAGCATACGA 469
E1         GTCATCAGCTA----AC---AGTCGTTA--AATTTTTATATATATTATTATAGCATACGA 614
27         GTCATCAGCTA----AC---AGTCGTTA--AATTTTTATATATATTATTATAGCATACGA 503

94p4       GACATCAGACAAGTCTT---AGACGTATTCAATTTTT-ATATATTTATTATAACATACGT 419
Am4        GACATCAGACAAGCCTT---AGACGTATTCAATTTTT-ATATATTTATTATAACATACGA 441
c6A        GACATCAGACAAGCCTT---AGACGTATTCAATTTTTTATATATTTATTATAGCATACGA 665
D4         GACATCAGACAAGCCTT---AGGCGTATTCAATTTT-TATATATTTATTATAACATACGT 448
12         GACATCAGACAAGTCTT---AGACGTATTCAATTTT-TATATATTTATTATAACATACGA 407
A3         GACATCAGACAAGCCTT---AGGCGTATTCAATTTT-TATATATTTATTATAACATACGT 399
L81        GACATCAGACAAGCCTT---AGGCGTATTCAATTTT-TATATATTTATTATAACATACGT 365
c2         GACATCAGACAAGCCTT---AGGCGTATTCAATTTT-TATATATTTATTATAACATACGT 365
           * *:* .*. :    :    **  .*::  ******  :::::: *******.******:
            <--PE1 -35
p6/4       TTTTTCAAAATCAATT-AAAAAATCAGCGTCAAAA-ACATAAGAATGGCTCAACCATGCG 505
923        TTTTTTAAAATCAAGCAAAAAAATCAGGGTAAAAA-ACACAAGAATTGCTCAACCGTGGG 773

bIL67      TTTTTCAAAATCAAGTAAAAAAATTAGGGTCAAAA-ACATAAGAATGGCTCAACCGTGGG 497
14         TTTTTCAAAATCAAGCGAAAAAATTAGGGTCAAA-AATAGAAGAATGGCTCAACCGTGGG 528
E1         TTTTTCAAAATCAAGCGAAAAAATTAGGGTCAAAAAATAGAAGAATGGCTCAACCGTGGG 674
27         TTTTTCAAAATCAAGCGAAAAAATTAGGGTCAAA-AATAGAAGAATGGCTCAACCGTGGG 562

94p4       TTTTTGAAAATCAAGTAAAAAAATTAGGGTAAAAA-ATAAAAGAAACGCTCAACTGTGGG 478
Am4        TTTTCCAAAATCAAGTAAAAAAATCAGGGTCAAAA-ATAGAAGAATGTCTCAACCGTGGG 500
c6A        TTTTTCAAAATCAAGTAAAAAAATTAGGGTCAAAA-ATAGAAGAATGGCTCAACCGTGGG 724
D4         TTTTTCAAAATCAAGTAAAAAAATCAGGGTCAAAA-ATACAAGAATGGCTCAACCACGCT 507
12         TTTTTCAAAATCAAGTAAAAAAATCAGGGTCAAAA-ATACAAGAATGGCTCAACCACGCT 466
A3         TTTTTCAAAATCAAGTAAAAAAATCAGGGTCAAAA-ATACAAGAATGGCTCAACCACGCT 458
L81        TTTTTCAAAATCAAGTAAAAAAATCAGGGTCAAAA-ATACAAGAATGGCTCAACCACGCT 424
c2         TTTTTCAAAATCAAGTAAAAAAATCAGGGTCAAAA-ATACAAGAATGGCTCAACCACGCT 424
           ****  ********   ******* ** **.***  * * *****:  ****** . *
```

```
                                                               3’ CT
p6/4      GATAGTCAGTATTATATTATTTTTTGGTTACAAATTATTTAATTAAATTGTAAATTATCT 565
923       AATAGTAGGTATTATATTATTTTTTGGTTACAAATTATTTAATCAAATTATAAACTATCT 833

bIL67     GATAGTCAGGAATATATTATTTTTTGGTTACAAATTATTTAATCAAATTGTAAAATATCT 557
14        AATAGTTAGGAATATATTATTTTTTGGTTACAAATTATTTAATCAAATTGTAAACTATCT 588
E1        AATAGTTAGGAATATATTATTTTTTGGTTACAAATTATTTAATCAAATTGTAAACTATCT 734
27        AATAGTTAGGAATATATTATTTTTTGGTTACAAATTATTTAATCAAATTGTAAACTATCT 622
94p4      AATAGTCAGGAATATATTATTTTTTAATTACAAATTATTTAATCAAATTGTAAACTATCT 538
Am4       AATAGTAAGGAATATATTATTTTTTGGTTACAAATTATTTAATCTAATTGTAAACTATCT 560
c6A       AATAGTCAGGAATATATTATTTTTTGGTTACAAATTATTTAATTAAATTGTAAACTATCT 784
D4        GATAGTTAGTATTATATTATTTTTTGGTTACAAATTATTTAATTAAATTGTAAACTATCT 567
12        GATAGTTAGTATTATATTATTTTTTGGTTACAAATTATTTAATTAAATTGTAAACTATCT 526
A3        GATAGTTAGTATTATATTATTTTTTGGTTACAAATTATTTAATTAAATTGTAAACTATCT 518
L81       GATAGTTAGTATTATATTATTTTTTGGTTACAAATTATTTAATTAAATTGTAAACTATCT 484
c2        GATAGTTAGTATTATATTATTTTTTGGTTACAAATTATTTAATTAAATTGTAAACTATCT 484
          .*****  .*  *:*************..****************  :****.**** *****

          AAATTTTTTGTAGGAAGCTTGGG 5’
p6/4      AAATTTTTTGTAGGTATAATAAAAGTTATAACTAAAAAGTAGTATGCTATAATAATAACA 625
923       AAATCTTTTGTTGGTATAATAAAAGTAAAAGTGAAAAAACAGTATGCTATAATAATACCA 893
            3’CTTTTGTTGGTATGAAGCTTGGG 5’27oriRHindIII
bIL67     AAATCTTTTGTTGGTATGATAAAAATAAAACTAAAAAAACAGTATGCTATAATAATACCA 617
14        AAATCTTTTGTTGGTATGATGAAACTAAAACTAAAAAAACAGTATGCTATAATAATACCA 648
E1        AAATCTTTTGTTGGTATGATGAAACTAAAACTAAAAAAACAGTATGCTATAATAATACCA 794
27        AAATCTTTTGTTGGTATGATGAAACTAAAACTAAAAAAACAGTATGCTATAATAATACCA 682
                              PL1  -35                    -10    3’ CA
94p4      AAATCTTTTGTAGGTATAATAAAAGTTATAACTAAAAATGGGTATGCTATAATAATAACA 598
Am4       AAATCTTTTGTTGGTATAATAAAAGTTATAACTAAAAATGGGTATGCTATAATAATAACA 620
c6A       AAATCTTTTGTTGGTATAATAAAAGTTATAACTAAAAATGGGTATGCTATAATAATACCA 844
D4        AAATCTTTTGTAGGTACAATAAAAGTTATAACTAAAAATGGGTATGCTATAATAATACCA 627
12        AAATCTTTTGTAGGTACAATAAAAGTCATAACTAAAAATGGGTATGCTATAATAATACCA 586
A3        AAATCTTTTGTAGGTACAATAAAAGTCATAACTAAAAATGGGTATGCTATAATAATACCA 578
L81       AAATCTTTTGTAGGTACAATAAAAGTTATAACTAAAAATGGGTATGCTATAATAATACCA 544
c2        AAATCTTTTGTAGGTACAATAAAAGTCATAACTAAAAATGGGTATGCTATAATAATACCA 544
          **** ******:****  .**.***  *  *:*    *****   .***************.**

p6/4      TAATAAACgagggaggTAA-AGACATGGCAGAAAAAAACATCTATTTTGTTA-------- 676
923       TAATCAATgagggaggTAAAAAGCATGGCAGAAAAAAACATCTATTTTGTTAATGATGAA 953
bIL67     TAATCAACgagggag--------------------------------------------- 632
14        TAATCAATgaggga---------------------------------------------- 662
E1        TAATCAATgagggaggTAAAAAGCATGGC------------------------------- 823
27        TAATCAATgagggagg-------------------------------------------- 698
           TAATCAAWGAGGGA—AAGCTTGGG 5’
94p4      TAATCAAAgaggg----------------------------------------------- 611
Am4       TAATCAATgagggaggTAAAAAGCATGGCAGAAAAAAACATCTATTTTGTTAATGATGAA 680
c6A       TAATCAATgagggaggTAA-AAGCATGGCAGAAAAAAACATCTATTTTGTTAATGATGAA 903
D4        TAATCAATgagggaggTAAAAAGCATGGCAGAAAAAAACATCTATTTGGTCAATGATGAA 687
12        TAATCAATgagggaggTAAA-AGCATGGCAGAAAAAAACATCTATTTTGTTAATGATGAA 645
A3        TAATCAATgagggaggTAA-AGACATGGCAGAAAAAAACATCTATTTTGTTAATGATG-- 635
L81       TAATCAATgagggaggTAAAAAGCATGGCAGAAAAAAACATCTATTTGGTCAATGATGAA 604
c2        TAATCAATgagggaggTAA-AGACATGGCAGAAAAAAACATCTATTTTGTTAATGATGAA 603
          ****.**  *****
                Shine-Dalgarno seq
```

Supplementary Table S1A. Host range of *Ceduovirus* phages sequenced in this study against a collection of industrial and environmental *L. lactis* strains.

	**Bacteriophages**											
**Strains**	14	27	94p4	E1	A3	L81	D4	12	Am4	p6/4	c2	bIL67
**Industrial strains**												
L. lactis subsp. *cremoris*												
IBB695-700, 1788-1792												
IBB1267*												
IBB1280*												
IBB1787*												
L. lactis subsp. *lactis*												
IBB732, 733, 743, 749, 754, 757, 758, 761, 763, 764,1784, 1796												
IBB750												
IBB752												
IBB753*												
IBB1219*												
L. lactis subsp. *lactis* biovar. *diacetylactis*												
IBB734-735, 741, 742, 744-748, 751 , 755, 756, 760												
IBB736, 737												
IBB738*												
IBB739*												
IBB762												
IBB1283*												
IBB1298*												
**Natural isolates**												
L. lactis subsp. *cremoris*												
IBB338												
IBB339, 342												
IBB704												
**Total no. of sensitive strains**	**4**	**4**	**5**	**11**	**2**	**3**	**7**	**1**	**1**	**1**	**0**	**0**
sensitive subsp. *cremoris*	0	0	1	4	2	2	4	1	0	0	0	0
sensitive subsp. *lactis*	0	0	1	2	0	0	2	0	1	0	0	0
sensitive biovar. diacetylactis	4	4	3	5	0	1	1	0	0	1	0	0

*The industrial starter strains used at the time of whey sample collection.
Gray-shaded boxes indicate clear plaques, white boxes –no plaques on respective strains.

Supplementary Table S1B. Host range of *Ceduovirus* phages sequenced in this study against laboratory *L. lactis* strains.

	**Bacteriophages**											
**Strains**	14	27	94p4	E1	A3	L81	D4	12	Am4	p6/4	c2	bIL67
L. lactis subsp. *cremoris*												
MG1363												
NZ9000												
L. lactis subsp. *lactis* biovar. diacetylactis												
IL1403												

Gray-shaded boxes indicate clear plaques, light gray boxes indicate turbid plaques and white boxes no plaques on respective strains.

Supplementary Table S2. Relative transformation efficiency of *L. lactis* strains with a phage ori+ test plasmid.

**Construct**	**MG1363 (*cremoris*)**	**IL1403 (*lactis*)**
pGHOST3 (competence control)	1.00E+00	1.00E+00
pUN121:catR:27ori (bIL67-group)	2.00E-04	1.93E-04
pUN121:catR:p6/4ori (923-group)	1.20E-02	3.00E-03
pUN121:catR:94p4ori (c2-group)	2.07E-02	1.82E-04
pUN121:catR:c2ori (control ori)	4.00E-03	5.68E-04

Transformation efficiency of the pG+host3 control vector (1 μg) was 1.5E+05 CFU μg^{-1} for MG1363 and 2.2E+06 CFU μg^{-1} for the IL1403 strain.

Supplementary Table S3. Summary list of host factors potentially influencing phage infection pattern. in association with genomic data of putative host range determinants.

	Phages											
	14	27	94p4	E1	A3	L81	D4	12	Am4	p6/4	c2	bIL67
**Host range data**												
recognized *L. lactis* CWPS type	B & U	B & U	B [T], C & U	B [T], C & U	C	C & U	C & U	C	B	B	B [T], C	B
infected subsp. of *L. lactis*	Diac.	Diac.	All	all	Crem.	Crem., Diac.	all	Crem.	Lac. Diac.	Diac.	Crem., Diac. [T]	Lac
infected subsp. of *L. lactis* reference strains	Diac.	Diac.	Crem., Diac. [T]	Crem., Diac. [T]	Crem.	Crem.	Crem.	Crem.	Diac.	Diac [T]	Crem., Diac. [T]	Diac.
no. of lysed strains	5	5	8	12	4	5	9	3	2	2	3	1
**Potential host range determinants**												
GP2 (AA)	397	397	401	416	415	415	415	418	397	413	415	397
GP3 (AA)	332	332	176	321	169	169	169	176	542	290	381	312
GP4 (AA)	628	628	628	638	638	638	638	638	628	638	638	628
GP8 (AA)	620	620	706	620	706	706	706	706	620	620	706	620
Tail length based on EM (mm)	92+/-5	98+/-6	101+/-8	96+/-6	81+/-6	101+/-5	95+/-4	87+/-9	82+/-5	92+/-3	nd	nd
GP14 (AA)	484	484	480	480	480	480	480	480	480	480	480	481

[T] turbid plaques; Diac., diacetylactis; Lac., *lactis*; Crem., *cremoris;* nd, not determined

References

1. Sievers, F.; Wilm, A.; Dineen, D.G.; Gibson, T.J.; Karplus, K.; Li, W.; Lopez, R.; McWilliam, H.; Remmert, M.; Söding, J. Fast, scalable generation of high-quality protein multiple sequence alignments using Clustal Omega. *Mol. Syst. Biol.* **2011**, *7*, 539.
2. Yoon, S.H.; Ha, S.M.; Lim, J.M.; Kwon, S.J.; Chun, J. A large-scale evaluation of algorithms to calculate average nucleotide identity. *Antonie Van Leeuwenhoek* **2017**, *110*, 1281–1286.
3. Lee, I.; Kim, Y.O.; Park, S.-Ch.; Chun, J. OrthoANI: An improved algorithm and software for calculating average nucleotide identity. *Int. J. System. Evol. Microbiol*. **2016**, *66*, 1100–1103.
4. Edgar, R.C. MUSCLE: Multiple sequence alignment with high accuracy and high throughput. *NAR* **2004**, *32*, 1792–1797.
